# Extravasation of *Borrelia burgdorferi* Across the Blood–Brain Barrier is an Extremely Rare Event

**DOI:** 10.1002/advs.202413199

**Published:** 2025-03-12

**Authors:** Linus Wang, Zikai Xia, Anjan Singh, Bhavna Murarka, Nicole Baumgarth, John N. Aucott, Peter C. Searson

**Affiliations:** ^1^ Institute for Nanobiotechnology Johns Hopkins University 3400 N Charles St Baltimore MD 21218 USA; ^2^ Department of Biomedical Engineering Johns Hopkins University 3400 N Charles St Baltimore MD 21218 USA; ^3^ Department of Materials Science and Engineering Johns Hopkins University 3400 N Charles St Baltimore MD 21218 USA; ^4^ Molecular and Cellular Biology Johns Hopkins University 3400 N Charles St Baltimore MD 21218 USA; ^5^ Department of Molecular Microbiology and Immunology Johns Hopkins University 615 N Wolfe St Baltimore MD 21205 USA; ^6^ Johns Hopkins Lyme Disease Research Center Johns Hopkins University 2360 Joppa Rd Timonium MD 21093 USA

**Keywords:** blood–brain barrier, dissemination, extravasation, tissue‐engineering, vector‐borne pathogens

## Abstract

Lyme disease, the most widespread tick‐borne disease in North America, is caused by the bacterium *Borrelia burgdorferi* (*Bb*). Approximately 10–15% of infections result in neuroborreliosis, common symptoms of which include headaches, facial palsy, and long‐term cognitive impairment. Previous studies of *Bb* dissemination focus on assessing *Bb* transmigration at static time points rather than analyzing the complex dynamic process of extravasation. Furthermore, current in vitro models lack crucial physiological factors such as flow, demonstrating a need for more robust models for studying *Bb* dissemination to understand its dynamics and mechanisms. Here, a 3D tissue‐engineered microvessel model is used and fluorescently‐labeled *Bb* is perfused to model vascular dissemination in non‐tissue‐specific (iEC) and brain‐specific (iBMEC) microvessels while acquiring time‐lapse images in real time. In iECs, extravasation involves two steps: adhesion to the endothelium and transmigration into the extracellular matrix, which can be modulated through glycocalyx degradation or inflammation. In contrast, *Bb* extravasation in iBMECs is an extremely rare event regardless of glycocalyx degradation or inflammation. In addition, circulating *Bb* do not induce endothelial activation in iECs or iBMECs, but induces barrier dysfunction in iECs. These findings provide a further understanding of *Bb* vascular dissemination.

## Graphical abstract

Lyme disease is caused by vascular dissemination of the bacteria *Borrelia*. In a 3D tissue‐engineered microvessel model, *Borrelia* extravasation across the blood–brain barrier is extremely rare, implying that neuroborreliosis is not caused via direct cytotoxicity. In contrast, *Borrelia* extravasate across non‐tissue specific microvessels which involves two steps: adhesion and transmigration, and can be modulated through glycocalyx degradation or inflammation.

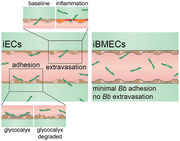

## 1 Introduction

Lyme disease is caused by infection of the bacterium *Borrelia burgdorferi* (*Bb*) and is the most prevalent tick‐borne disease in North America.^[^ ^1^, ^2^, ^3^ ^]^ The incidence of Lyme disease has steadily increased over the past 30 years, and is projected to continue growing by as much as 20% in the coming decades, partially due to climate change.^[^ ^4^, ^5^, ^6^ ^]^ Based on insurance records from 2000 to 2018, the Centers for Disease Control and Prevention recently estimated that there are approximately 476000 new cases of Lyme disease every year in the United States.^[^ ^7^, ^8^ ^]^

After the bite from an infected tick, *Bb* is inoculated into the skin and begins to proliferate and migrate in the local tissue where it can intravasate into circulation or the lymph system to infect distant organs and tissues,^[^ ^1^, ^3^ ^]^ particularly the skin, joints, heart, and central nervous system.^[^ ^9^ ^]^ Roughly 10–15% of cases of Lyme disease lead to infection of the central nervous system, or neuroborreliosis, common symptoms of which include headaches, meningitis, facial palsy, and radiculoneuritis.^[^ ^1^, ^3^, ^10^ ^]^ Despite these well‐known symptoms, the pathogenesis of neuroborreliosis and associated neural dysfunctions remains unclear. A central question is whether *Bb* can cross the blood–brain barrier (BBB) and directly infect neurons and glial cells, or whether neuroborreliosis is the result of indirect mechanisms such as the local generation of neurotoxic compounds or autoimmune reactions.^[^ ^11^, ^12^ ^]^ Recent intravital microscopy (IVM) studies in mice show that *Bb* are able to colonize the dura mater but are rarely found in the brain parenchyma, suggesting that *Bb* cannot cross the BBB.^[^ ^13^, ^14^ ^]^

Most of our knowledge of *Bb* dissemination comes from analysis of tissue samples in mouse models, e.g. enumeration of bacteria at the inoculation site and in other tissues and organs. However, the processes associated with dissemination are inherently dynamic, and hence IVM studies in mouse models have been crucial in beginning to unravel mechanistic details.^[^ ^13^, ^15^, ^16^, ^17^, ^18^, ^19^, ^20^, ^21^, ^22^, ^23^, ^24^, ^25^ ^]^ These reports have been complemented by studies of *Bb* adhesion in other in vitro models, such 2D flow chambers and Transwells,^[^ ^26^, ^27^, ^28^, ^29^ ^]^ 3D migration studies,^[^ ^30^ ^]^ and membrane feeding assays.^[^ ^31^ ^]^ However, these approaches have limited physiological relevance due to the absence of critical factors such as flow or the inability to observe transmigration across 2D plated endothelial cells on glass or plastic substrates. As such, we have developed a 3D tissue‐engineered microvessel that incorporates these factors crucial for pathogen dissemination to model *Bb* extravasation.

To determine how *Bb* extravasate from circulation and whether *Bb* can cross the BBB, we imaged *Bb*‐microvessel interactions in non‐tissue‐specific and brain‐specific tissue‐engineered microvessel models perfused with GFP‐labeled *Bb* (B31‐A3 strain). Microvessels were formed with isogenic stem cell‐derived endothelial cell lines: induced endothelial cells (iECs) as a non‐tissue‐specific control, and induced brain microvascular endothelial cells (iBMECs) to model the BBB. We show that *Bb* extravasation across the BBB is extremely rare, consistent with native infections in mouse models and human autopsy samples. This also suggests that the pathogenesis of neuroborreliosis is not via direct cytotoxicity. The overall process of extravasation occurs through two separate steps: 1) the initial adhesion to endothelial cells, and 2) transmigration across the endothelial barrier into the surrounding ECM. Removal of components of the glycocalyx (heparan sulfate, sialic acid) reduces adhesion, while chronic inflammation promotes higher transmigration rates. In addition, *Bb* perfusion does not induce endothelial activation in iEC or iBMEC microvessels, but does induce barrier disruption in iEC microvessels. These results suggest that symptoms associated with neuroborreliosis do not arise during the sub‐acute phase of infection.

## 2 Results

### 2.1 Development of *Borrelia burgdorferi* Extravasation Model

**Figure 1:**
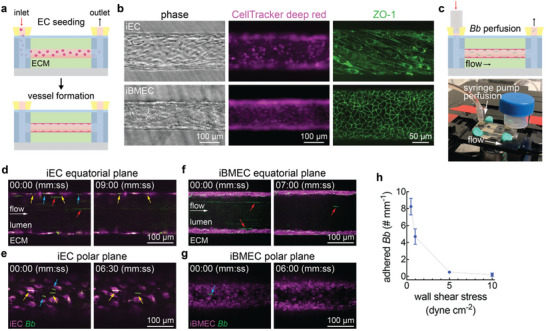
Perfusion of *Borrelia burgdorferi* (*Bb*) in induced endothelial cell (iEC) and induced brain microvascular endothelial (iBMEC) microvessels. (a) Schematic illustration of endothelial cell (EC) seeding into a 150 µm diameter channel in a collagen type I extracellular matrix (ECM) to form a microvessel model. (b) Representative phase and fluorescence images of iEC and iBMEC microvessels stained with CellTracker deep red. Both cell lines also express zona occludens‐1 (ZO‐1) tagged with green fluorescent protein (GFP), which were imaged with confocal microscopy. Representative images were produced through maximum intensity projections over 40 µm. (c) Schematic illustration and photograph of the *Bb* perfusion setup with a syringe pump on a microscope stage in a live cell chamber for real‐time imaging. (d) Fluorescence image of *Bb* perfusion at 1 dyne cm^−2^ in a CellTracker deep red stained iEC microvessel focused at the equatorial plane. Yellow arrows denote adhered *Bb*, blue arrows denote *Bb* that subsequently detached after 9 min of flow, and red arrows denote *Bb* flowing in the lumen of the microvessel that did not interact with the microvessel. (e) Fluorescence image of *Bb* perfusion at 1 dyne cm^−2^ in a CellTracker deep red stained iEC microvessel focused at the polar plane. Yellow arrows denote adhered *Bb* and blue arrows denote *Bb* that subsequently detached after 6.5 min of flow. (f) Fluorescence image of *Bb* perfusion at 1 dyne cm^−2^ in a CellTracker deep red stained iBMEC microvessel focused at the equatorial plane. Red arrows denote *Bb* flowing in the lumen of the microvessel that did not interact with the microvessel. (g) Fluorescence image of *Bb* perfusion at 1 dyne cm^−2^ in a CellTracker deep red stained iBMEC microvessel focused at the polar plane. Blue arrows denote *Bb* that subsequently detached after 6 min of flow. (h) Number of adhered *Bb* in iEC microvessels after 1 hour of perfusion at 10, 5, 1, and 0.5 dyne cm^−2^ (n = 3). Microvessels were perfused with *Bb* for 1 hour and the number of firmly adhered *Bb* was then counted.

Tissue‐engineered microvessels were formed by seeding induced endothelial cells (iECs) or induced brain microvascular endothelial cells (iBMECs) into a 150 µm diameter channel located in a type I collagen matrix (**Figures** **1**a,b and S1, Supporting Information). To identify a co‐culture medium to support both bacteria and endothelial cells, *Bb* were inoculated in various mixtures of BSK‐H and endothelial cell (EC) medium (Figure S2a–e, Note S1, Supporting Information). *Bb* exhibited comparable expression levels of key genes (*bbk32*, *ospC*, *bb0323*), similar levels of adhesion onto fibronectin coated glass surfaces, and no distinguishable differences in motility in a 3D collagen gel after 24 h of conditioning in medium from BSK‐H alone to 1:1 BSK‐H:EC media (Figures S2f–m and S3, Supporting Information). At the same time, iEC and iBMEC microvessels maintained barrier function as demonstrated through permeability assays and structurally intact tight junctions, with no significant endothelial activation at 24 h after perfusion with medium in the same composition range (Figure S4, Note S2, Supporting Information). Therefore, for all experiments reported here, microvessels were matured for 24 h in EC medium and then conditioned in co‐culture medium (1:1 BSK‐H:EC medium) for 24 h prior to perfusion with *Bb* in co‐culture medium (Figure S5a, Supporting Information).

After microvessel maturation and conditioning with co‐culture medium*, Bb* were perfused at a density of 5 × 10^6^ cells mL^−1^ with a syringe pump at a constant flow rate (wall shear stress) (Figure 1c). Time‐lapse images of *Bb‐*iEC microvessel interactions were acquired at the equatorial and polar planes (Figure 1d–g; Movies S1–S3, Supporting Information). To determine the effect of flow on adhesion, microvessels were perfused with *Bb* at shear stresses up to 10 dyne cm^−2^. *Bb* adhesion increased with decreasing shear stress (Figure 1h), and all subsequent experiments were performed at 1 dyne cm^−2^ to recapitulate the physiological shear stress in post‐capillary venules which is generally considered to be the primary site of extravasation of pathogens and immune cells.^[^ ^9^, ^32^, ^33^, ^34^, ^35^, ^36^ ^]^

### 2.2 *Borrelia burgdorferi* Interactions with iEC and iBMEC Microvessels

**Figure 2:**
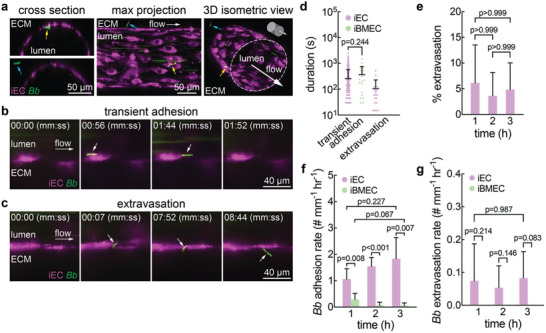
*Borrelia burgdorferi* (*Bb*) adhesion and extravasation from induced endothelial cell (iEC) and induced brain microvascular endothelial cell (iBMEC) microvessels. (a) Representative cross section, maximum intensity projection, and 3D reconstruction confocal images of *Bb* adhered to CellTracker deep red stained iEC microvessels. *Bb* are shown to be adhered to the lumen side of the microvessels (yellow arrow) or extravasated into the ECM (blue arrow). (b) Fluorescence image sequence of a *Bb* transiently adhered in an iEC microvessel stained with CellTracker deep red. A *Bb* adheres on the lumen side of the microvessel at t = 56 seconds, remains adhered at t = 1 minute 44 seconds, and detaches at approximately t = 1 minute 52 seconds. (c) Fluorescence image sequence of a *Bb* extravasating from an iEC microvessel stained with CellTracker deep red. A *Bb* adheres on the lumen side of the microvessel and begins transmigration at t = 7 seconds, continues to transmigrate at t = 7 minute 52 seconds, and completes extravasation at t = 8 minute 44 seconds where it is fully in the ECM. (d) Duration of transient adhesion and extravasation events. No extravasation events in iBMEC microvessels were recorded. iEC: transient adhesion n = 437, extravasation n = 19 over 5 independent microvessels. iBMEC: transient adhesion n = 19, extravasation n = 0 over 6 independent microvessels. (e) Percent of adhered *Bb* that extravasated during each hour of perfusion in iEC microvessels. Percentages are calculated based on total known outcomes of *Bb*‐microvessel interactions. n = 5. (f) *Bb* adhesion rate normalized by microvessel length during each hour of perfusion in iEC or iBMEC microvessels. Adhesion is counted if a *Bb* remained in focus at the same location between two frames of imaging. Images were acquired every 15 seconds. iEC microvessels, n = 5. iBMEC microvessels, n = 6. (g) *Bb* extravasation rate normalized by microvessel length during each hour of perfusion in iEC microvessels. No extravasation events were observed across 6 iBMEC microvessels. iEC microvessels, n = 4. iBMEC microvessels, n = 6.

To visualize and quantify *Bb* adhesion and extravasation, microvessels were perfused with *Bb* for 3 h (Movies S1–S4¸ Supporting Information). Microvessels were first perfused with either fluorescently labeled 2 MDa or 10 kDa dextran to confirm barrier integrity and the absence of focal leaks. We then perfused *Bb* at a density of 5 × 10^6^ mL^−1^, and observed *Bb* adhesion to the microvessel lumen as well as extravasation into the surrounding extracellular matrix (ECM) (**Figure** **2**a, Movie S4, Supporting Information). To quantify *Bb*‐microvessel interactions, time‐lapse images were acquired every 15 s to measure the *Bb* adhesion rate during each hour of perfusion. To include an adhesion event for quantification, the *Bb* remained in focus at the same location for at least two frames.

We observed two types of *Bb*‐microvessel interactions: transient adhesion and extravasation (Figure 2b,c, and Figure S6, Supporting Information). Transient adhesion was characterized by *Bb* attachment and subsequent detachment in the direction of flow (Figure 2b; Movie S6, Supporting Information). The mean durations of transient adhesion in iEC and iBMEC microvessels were 259 ± 320 and 368 ± 390 s respectively (Figure 2d). Although the residence time was longer for iBMECs, the difference was not statistically significant (p = 0.244). Extravasation was characterized by initial adhesion followed by transmigration across the endothelium into the surrounding matrix (Figure 2c; Movie S7, Supporting Information). The mean time for extravasation, starting from the initial adhesion, was 105 ± 126 s in iEC microvessels. Over five independent iEC microvessels, approximately 5% of adhered *Bb* extravasated (Figure 2e). No *Bb* transmigration events were observed across six independent iBMEC microvessels.

The adhesion rate in iEC microvessels was significantly higher than in iBMECs (Figure 2f), but did not increase significantly over 3 h of perfusion. *Bb* extravasated from iEC microvessels at an average rate of 0.08 mm^−1^ h^−1^ and did not significantly increase over 3 h (Figure 2g). *Bb* that remained attached to the endothelium at the end of imaging were not included in the analysis (Figure S6, Note S3, Movie S8, Supporting Information). Although we did not visualize *Bb* extravasation across iBMECs, we observed one *Bb* in the ECM across six iBMEC microvessels and 18 h of imaging (Figure S7b, Supporting Information).

### 2.3 Influence of the Glycocalyx on *Borrelia burgdorferi*‐Microvessel Interactions

Since the glycocalyx plays a role in inflammation, leukocyte adhesion, and BBB permeability,^[^ ^37^, ^38^, ^39^ ^]^ we assessed the influence of the glycocalyx on *Bb*‐microvessel interactions. To induce glycocalyx degradation, we treated microvessels with a combination of 50 mU mL^−1^ of heparinase III and 20 mU mL^−1^ of sialidase for 48 h, starting immediately following microvessel seeding. Glycocalyx degradation was confirmed through immunostaining for heparan sulfate and wheatgerm agglutinin, along with immune cell adhesion assays (Figure S8a–f¸ Supporting Information). From image analysis, we estimated that the remaining heparan sulfate and sialic acid residues were approximately 65% and 40%, respectively, of their initial values (Figure S8c,d, Supporting Information).

**Figure 3:**
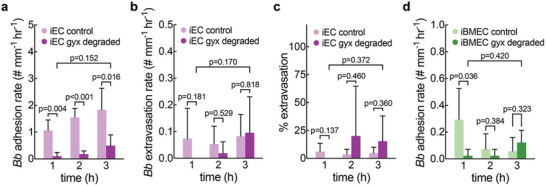
Effect of glycocalyx degradation on *Borrelia burgdorferi*‐microvessel interactions. (a) *Bb* adhesion rate normalized by microvessel length during each hour of perfusion in glycocalyx degraded or control iEC microvessels. Glycocalyx degraded n = 5. Control n = 5. (b) *Bb* extravasation rate normalized by microvessel length during each hour of perfusion in glycocalyx degraded or control iEC microvessels. Glycocalyx degraded n = 5. Control n = 6. (c) Percent of *Bb*‐microvessel adhesions that resulted in extravasation during each hour of perfusion in glycocalyx degraded or control iEC microvessels. Percentages are calculated based on total known outcomes of *Bb*‐microvessel interactions. Glycocalyx degraded n = 5. Control n = 6. (d) *Bb* adhesion rate normalized by microvessel length during each hour of perfusion in glycocalyx degraded or control iBMEC microvessels. Glycocalyx degraded n = 4. Control n = 6.

In iEC microvessels, glycocalyx degradation decreased the *Bb* adhesion rate and the number of adherent *Bb* by about four‐fold (**Figure** **3**a; Figure S8g, Supporting Information). However, there was no significant change in the *Bb* extravasation rate, despite a large variability in the data (Figure 3b,c). This wide variance is likely due to the reduction in *Bb* adhesion and as a result, any instance of *Bb* extravasation introduces large variability in the percentage of adhered *Bb* that extravasate. In iBMEC microvessels, there was no significant difference in the *Bb* adhesion rate except during the first hour of perfusion (Figure 3d). No extravasated *Bb* were observed across four microvessels, and all *Bb*‐iBMEC interactions were transient adhesion events.

### 2.4 Influence of Inflammation on *Borrelia burgdorferi*‐Microvessel Interactions

We next assessed whether chronic inflammation affected *Bb*‐microvessel interactions. To model inflammation, we perfused microvessels with 5 ng mL^−1^ of TNF‐α for 12 h. We confirmed that TNF‐α induced inflammation through immune cell adhesion and solute permeability assays (Figure S9, Supporting Information). Perfusion with TNF‐α resulted in an increase in expression of intercellular adhesion molecule (ICAM‐1) and an increase in immune cell adhesion frequency in both iEC and iBMEC microvessels (Figure S9c–f¸ Supporting Information). In iEC microvessels, TNF‐α resulted in an increase in permeability to 2 MDa, however, in iBMEC microvessels there was no change in permeability to 10 kDa (Figure S9g,h, Supporting Information).

**Figure 4:**
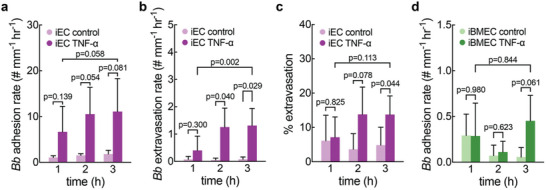
Effect of TNF‐α on *Borrelia burgdorferi*‐microvessel interactions. (a) *Bb* adhesion rate normalized by microvessel length during each hour of perfusion in TNF‐α treated or control iEC microvessels. TNF‐α n = 3. Control n = 5. (b) *Bb* extravasation rate normalized by microvessel length during each hour of perfusion in TNF‐α treated or control iEC microvessels. TNF‐α n = 4. Control n = 6. (c) Percent of *Bb*‐microvessel adhesions that resulted in extravasation during each hour of perfusion in TNF‐α treated or control iEC microvessels. Percentages are calculated based on total known outcomes of *Bb*‐microvessel interactions. TNF‐α n = 4. Control n = 5. (d) *Bb* adhesion rate normalized by microvessel length during each hour of perfusion in TNF‐α treated or control iBMEC microvessels. TNF‐α n = 4. Control n = 6.

For iEC microvessels, TNF‐α perfusion increased the *Bb* adhesion rate by approximately eight‐fold, but was not statistically significant (**Figure** **4**a). In addition, the extravasation rate significantly increased during the second and third hours of perfusion (Figure 4b). There was a significant increase in the percentage of adhesion events that resulted in extravasation during the third hour of perfusion (Figure 4c).

For iBMEC microvessels, TNF‐α treatment did not affect the *Bb* adhesion rate, and no extravasation events were recorded (Figure 4d). The *Bb* adhesion rate remained relatively constant over 3 h of perfusion. Similar to the control condition (no TNF‐α), one *Bb* was observed in the ECM after the third hour of perfusion across four independent microvessels (Figure S9l, Supporting Information). However, no extravasation events were recorded, and all interactions were of transient adhesions.

### 2.5 Effect of Circulating *Borrelia burgdorferi* on Endothelial Activation

**Figure 5:**
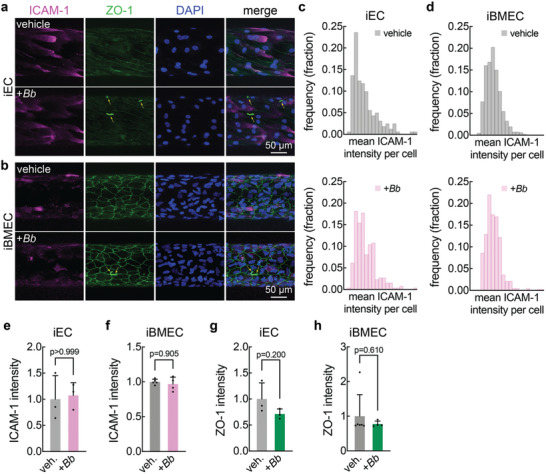
ICAM‐1 expression in induced endothelial cell (iEC) and induced brain microvascular endothelial cell (iBMEC) microvessels perfused with *Borrelia burgdorferi* (*Bb*). (a) Representative maximum intensity projection confocal images of ICAM‐1 (magenta) immunostained iEC microvessels with ZO‐1 (green) and DAPI (blue) after 3 h of perfusion with *Bb* or vehicle. (b) Representative maximum intensity projection confocal images of ICAM‐1 (magenta) immunostained iBMEC microvessels with ZO‐1 (green) and DAPI (blue) after 3 h of perfusion with *Bb* or vehicle. (c) Histogram of individual iEC cell ICAM‐1 fluorescence after 3 h of perfusion with *Bb* or vehicle. +*Bb* n = 161 cells across 3 independent microvessels. Vehicle n = 259 individual cells across 3 independent microvessels. (d) Histogram of individual iBMEC cell ICAM‐1 fluorescence after 3 h of perfusion with *Bb* or vehicle. +*Bb* n = 418 cells across 4 independent microvessels. Vehicle n = 518 individual cells across 6 independent microvessels. (e) Mean ICAM‐1 fluorescence in iEC microvessels from maximum intensity projection confocal images over 50 µm after 3 h of perfusion with *Bb* or vehicle. +*Bb* n = 3. Vehicle n = 3. (f) Mean ICAM‐1 fluorescence in iBMEC microvessels from maximum intensity projection confocal images over 50 µm after 3 h of perfusion with *Bb* or vehicle. +*Bb* n = 4. Vehicle n = 6. (g) Mean ZO‐1 fluorescence in iEC microvessels from maximum intensity projection confocal images over 50 µm after 3 h of perfusion with *Bb* or vehicle. +*Bb* n = 3. Vehicle n = 3. (h) Mean ZO‐1 fluorescence in iBMEC microvessels from maximum intensity projection confocal images over 50 µm after 3 h of perfusion with *Bb* or vehicle. +*Bb* n = 4. Vehicle n = 6.

**Figure 6:**
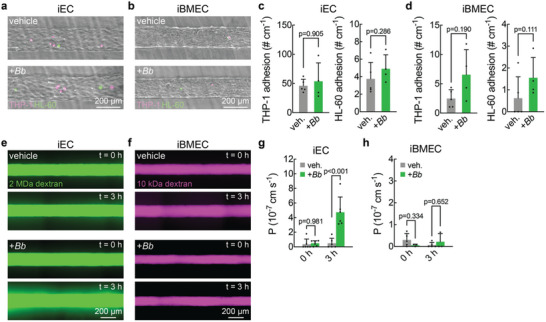
Effect of circulating *Borrelia burgdorferi* (*Bb*) on microvessel function. (a) Fluorescence images of adhered immune cells (THP‐1 in magenta, HL‐60 in green) overlaid on phase images of iEC microvessels. Immune cells were perfused at a density of 1 × 10^6^ cells mL^−1^ for 10 min followed by 30 min of vehicle to remove weakly adherent cells. (b) Fluorescence images of adhered immune cells (THP‐1 in magenta, HL‐60 in green) overlaid on phase images of iBMEC microvessels. Immune cells were perfused at a density of 1 × 10^6^ cells mL^−1^ for 10 min followed by 30 min of vehicle to remove weakly adherent cells. (c) THP‐1 and HL‐60 adhesion in iEC microvessels after 3 h of perfusion with *Bb* or vehicle. +*Bb* n = 5. Vehicle n = 4 (d) THP‐1 and HL‐60 adhesion in iBMEC microvessels after 3 h of perfusion with *Bb* or vehicle. +*Bb* n = 5. Vehicle n = 4. (e) Fluorescence images of iEC microvessels perfused with 2 MDa dextran before and after 3 h of perfusion with *Bb* or vehicle. (f) Fluorescence images of iBMEC microvessels perfused with 10 kDa dextran before and after 3 h of perfusion with *Bb* or vehicle. (g) Permeability of 2 MDa dextran in iEC microvessels before and after 3 h of perfusion with *Bb* or vehicle. +*Bb* n = 6. Vehicle n = 6. (h) Permeability of 10 kDa dextran in iBMEC microvessels before and after 3 h of perfusion with *Bb* or vehicle. +*Bb* n = 5. Vehicle n = 3.

To determine whether *Bb* perfusion induced endothelial activation, we assessed changes in pathways associated with initiating an immune response. This included measuring the level of ICAM‐1 expression in individual ECs and the frequency of immune cell recruitment after 3 h of perfusion with vehicle or *Bb* (**Figures** **5** and **6**a‐d). The fluorescence intensity for individual cells was determined through maximum intensity projections and using the signal from the endogenous ZO‐1 fluorescence to identify cell‐cell boundaries. The distributions of ICAM‐1 expression were similar for iECs and iBMECs following perfusion with vehicle or *Bb* (Figure 5c,d). This was confirmed from comparison of the mean intensity between conditions (*p* > 0.999 for iECs and p = 0.905 for iBMECs) (Figure 5e,f). In addition, we assessed the fluorescence intensity of the tight junction adaptor protein ZO‐1 (Figure 5g,h), and found no significant changes in fluorescence intensity (*p* = 0.200 for iECs and *p* = 0.610 for iBMECs). Both iECs and iBMECs perfused with *Bb* or vehicle exhibited well‐formed tight junctions with no apparent defects (Figure 5a,b).

To assess changes in immune cell recruitment, we perfused monocyte‐like (THP‐1) and neutrophil‐like (HL‐60) cells and counted the number of firmly adhered cells following washing out (Figure 6a,b). *Bb* perfusion did not cause a statistically significant increase in immune cell adhesion for either iEC or iBMEC microvessels (Figure 6c,d). Together these results suggest that *Bb* do not independently induce EC activation at this concentration and duration.

To determine the influence of *Bb* in circulation on microvessel barrier function, we assessed permeability through perfusion with 2 MDa dextran in iEC microvessels and 10 kDa dextran in iBMEC microvessels (Figure 6e,f). Note that the molecular weights of the solutes were matched to give similar permeabilities under control conditions. In iEC microvessels, the permeability of 2 MDa dextran increased 9.8‐fold from 0.48 × 10^−7^ to 4.75 × 10^−7^ cm s^−1^ after *Bb* perfusion compared to vehicle (p < 0.001) (Figure 6g). However, no significant difference in permeability was observed between vehicle and *Bb* perfusion conditions in iBMEC microvessels (Figure 6h).

### 2.6 Summary of Results

Through perfusing *Bb* in 3D tissue‐engineered microvessel models, we found that *Bb* adheres and extravasates from non‐tissue‐specific iEC microvessels at a much higher frequency than from brain‐specific iBMEC microvessels. In iECs, glycocalyx degradation decreased *Bb* adhesion, while inflammation induced by treatment with TNF‐α increased *Bb* extravasation. However, in iBMECs, neither glycocalyx degradation nor inflammation affected *Bb*‐microvessel interactions. After 3 h of *Bb* perfusion, there was no evidence of endothelial activation in either iEC or iBMEC microvessels through assessment of ICAM‐1 expression or immune cell adhesion. However, barrier dysfunction was induced in iEC microvessels.

## 3 Discussion

In the human brain, post‐capillary venules (typically < 200 µm in diameter) are thought to be the preferential sites of immune surveillance, transport of plasma proteins, and extravasation of pathogens due to the lower shear stress compared to arterioles or capillaries.^[^ ^34^, ^36^, ^40^, ^41^, ^42^ ^]^ During perfusion with *Bb*, adhesion in non‐tissue‐specific iEC microvessels decreased with increasing wall shear stress, confirming that flow conditions play an important role in dissemination (Figure 1f).^[^ ^43^ ^]^ Over 3 h of *Bb* perfusion at a wall shear stress of 1 dyne cm^−2^, we observed multiple extravasation events in iEC microvessels, suggesting that chemoattractants or additional components are not needed in the ECM to facilitate endothelial transmigration in our model (Figure 2c, Movie S7).

### 3.1 *Borrelia burgdorferi*‐Microvessel Interactions in iBMECs Relevant to Neuroborreliosis

In iBMEC microvessels, *Bb* showed a low rate of transient adhesion with an average number of adherent *Bb* about 80‐fold lower compared to iEC microvessels after 3 h of perfusion (Figure 2f). Extravasation of *Bb* was an extremely rare event in iBMEC microvessels: over six independent microvessels, we observed one *Bb* in the ECM (Figure S7b, Supporting Information). Based on a *Bb* concentration of 5 × 10^6^ mL^−1^, a flow rate of 2 µL min^−1^, and a total perfusion time of 3 h, this corresponds to ≈1 in 10^7^ *Bb*. These results are in agreement with prior studies that report very little or no *Bb* extravasation in various in vitro BBB models and in vivo models.^[^ ^13^, ^14^, ^44^, ^45^, ^46^ ^]^ Recent IVM studies in mice indicate that *Bb* are able to colonize the dura mater but are rarely found in the brain parenchyma.^[^ ^13^, ^14^ ^]^ Similarly, in non‐human primates, *Bb* are found mainly in the leptomeninges and have been shown to induce inflammation, while intact *Bb* in the cerebrum are seldom reported.^[^ ^47^, ^48^, ^49^ ^]^ Human autopsy reports also show that the majority of *Bb* in the brain are associated with the leptomeninges, the ependymal region of the fourth ventricle, or the brain stem.^[^ ^50^, ^51^, ^52^, ^53^, ^54^ ^]^ Although some *Bb* have been found in the parenchyma either as individual spirochetes or as biofilms, it is extremely rare and unpredictable.^[^ ^55^, ^56^ ^]^ As such, it is possible that *Bb* infect the brain via an alternative pathway such as through the blood‐cerebrospinal fluid barrier or along peripheral nerves, but any level of inflammation that may be induced in the brain following infection is still insufficient to promote *Bb* extravasation across the BBB.^[^ ^12^, ^57^, ^58^, ^59^ ^]^ Our results suggest that the tight junctions in iBMECs are effective in excluding *Bb* from the brain even at very high concentrations and under the inflammation conditions explored here. Therefore, our iEC and iBMEC models are able to recapitulate certain key differences in *Bb* transmigration in the brain and other tissues.

### 3.2 Comparison of iECs and iBMECs

Adhesion of *Bb* to iEC microvessels was much more common than in iBMEC microvessels, and resulted in more extravasation events (Figure 2f,g). Brain endothelial cells are one of the primary components of the BBB, which tightly controls the brain microenvironment by regulating transport of various chemicals and restricting the entry of harmful pathogens.^[^ ^60^, ^61^, ^62^ ^]^ As such, brain endothelial cells have enriched expression of tight junction proteins which help to maintain the high barrier function of the BBB.^[^ ^63^, ^64^, ^65^ ^]^ This may explain why *Bb* extravasation across iBMECs is much rarer than in iECs which model non‐tissue‐specific microvessels.

Also in contrast to iBMEC microvessels, glycocalyx degradation decreased the *Bb* adhesion rate and frequency in iECs (Figure 3a; Figure S8g, Supporting Information), suggesting that the glycocalyx, particularly heparan sulfate and sialic acid residues, facilitate *Bb* adhesion.^[^ ^66^, ^67^, ^68^, ^69^ ^]^ This is supported by previous 2D cell culture studies demonstrating that heparan sulfate degradation decreases *Bb* adhesion to human epithelial and endothelial cells by 25 to 80%.^[^ ^70^, ^71^, ^72^, ^73^, ^74^ ^]^ Though the *Bb*‐endothelial cell adhesion mechanism is not well known, one proposed model suggests that *Bb* express adhesin proteins such as BBK32 that bind to glycosaminoglycans and facilitate the initial interactions with endothelial cells prior to extravasation.^[^ ^9^, ^75^ ^]^ However, in iBMEC microvessels, glycocalyx degradation did not significantly affect *Bb* adhesion or extravasation except during the first hour of perfusion, likely because adhesion was extremely rare even under baseline conditions compared to iECs. Furthermore, TNF‐α treatment significantly increased *Bb* extravasation in iECs but not in iBMECs (Figure 4b Figure S9j, Supporting Information). This difference could be explained by a more robust response to activation via TNF‐α in iECs than iBMECs, as shown by the four‐fold increase in ICAM‐1 expression in iECs compared to less than a two‐fold increase in iBMECs (Figure S9c,d, Supporting Information). These differences highlight how *Bb* interactions differ based on the endothelial cell type, and the importance of using tissue‐specific vasculature for studying dissemination into specific tissues or organs such as the joint, heart, or brain.^[^ ^45^ ^]^

Despite these key differences, there were several similarities in *Bb*‐microvessel interactions between iECs and iBMECs. First, the *Bb* adhesion and extravasation rates remained constant over 3 h of perfusion, which is consistent with previous studies demonstrating that 3 h of infection is not sufficient to facilitate increased rates of *Bb* transmigration.^[^ ^19^ ^]^ Furthermore, a significant majority of *Bb* were transiently adherent and continued to perfuse through the microvessel. This suggests that after *Bb* intravasation into the vasculature, most *Bb* remain in circulation, where they may be taken up by phagocytic cells in circulation or by Kupffer and invariant natural killer T cells in the liver to prevent further dissemination.^[^ ^20^, ^21^ ^]^ Perfusion with *Bb* did not increase the mean level of ICAM‐1 expression per cell in both iEC and iBMEC microvessels (Figure 5), and individual cells that had at least one adhered *Bb* did not have significantly elevated expression of ICAM‐1 (Figure 10c–f). This suggests that *Bb* adhesion is a stochastic process, and that *Bb* do not preferentially adhere to cells with high ICAM‐1 expression.

### 3.3 Comparison with Other Models of *Borrelia* Perfusion and Extravasation

Current studies of *Bb* extravasation have been primarily limited to IVM in the dermis (flank and ear) and knee joints of mouse models following intravenous injection of *Bb*.^[^ ^15^, ^17^, ^18^, ^19^, ^22^, ^76^ ^]^ A common alternative is to use in vitro models such as 2D flow chambers, but these models have limited applications such as the inability to observe *Bb* extravasation.^[^ ^26^, ^27^, ^28^ ^]^ In contrast, the 3D tissue‐engineered microvessel platform used in this study provides a more physiologically relevant model that can be used to observe the complete extravasation process. Despite these various approaches to studying *Bb* extravasation, there are several notable similarities across these models.

In all models, shear stress plays an important role in regulating *Bb* adhesion. IVM studies largely focus on imaging post‐capillary venules (PCVs) and report observations of *Bb* interactions with capillaries and larger veins but not arterioles which experience higher shear forces.^[^ ^15^, ^16^, ^19^, ^75^ ^]^ Likewise, in vitro models with *Bb* are primarily perfused at shear stresses typical of PCVs (0.5–3.0 dyne cm^−2^), since *Bb* adhesion significantly decreases at higher shear stresses (Figure 1f).^[^ ^26^, ^27^, ^28^ ^]^

Under these flow conditions, *Bb*‐microvessel interaction rates were similar in in vivo IVM studies and in vitro 3D microvessels reported here. In one of the first IVM studies of extravasation, *Bb*‐blood vessel interactions were classified as “stationary adhesions” if a *Bb* adhered to a blood vessel and did not translate for at least 20 s.^[^ ^15^ ^]^ In the 3D microvessel model used here, images were acquired every 15 s and *Bb* interactions were only counted if it remained adhered for at least two frames of imaging. As such, *Bb* stationary adhesion rates were compared. After normalizing imaging duration, microvessel length and *Bb* density, IVM studies reported *Bb* stationary adhesion rates of ≈0.8 interactions h^−1^ mm^−1^ at 10^6^ *Bb* mL^−1^.^[^ ^16^, ^26^, ^28^ ^]^ This is similar to the adhesion rate observed in our iEC microvessel model of 0.3 interactions h^−1^ mm^−1^ at 10^6^ *Bb* mL^−1^ averaged across 3 h of *Bb* perfusion (Figure 2f).

In IVM studies, a little over 1% of *Bb* that adhered or dragged along the endothelium escaped from the vasculature,^[^ ^16^ ^]^ similar to our 3D iEC microvessel model where 5–10% of adhered *Bb* extravasated (Figure 2e). While a study involving a 2D flow chamber coupled with a Transwell membrane also reported 3–5% of *Bb* extravasating, this was calculated based on the total amount of *Bb* perfused rather than of those adhered to endothelial cells.^[^ ^77^ ^]^ Therefore, the fraction of extravasating *Bb* following adhesion in this model is likely much higher than in IVM studies. This highlights how our 3D microvessel which incorporates key physiological conditions can recapitulate similar results found in vivo. Furthermore, in all models of dissemination, the significant majority of *Bb* interactions with the endothelium are of rapid transient adhesions, while extravasation occurs much less frequently.

### 3.4 Two‐Step Mechanism of *Borrelia burgdorferi* Extravasation

Through perfusing *Bb* through iEC microvessels, we found that glycocalyx degradation only affected *Bb* adhesion, while TNF‐α treatment only affected *Bb* extravasation. This implies that the overall process of *Bb* extravasation is governed by two distinct steps, each with their own mechanism: the initial adhesion to the endothelium, and the subsequent transmigration into the ECM.

Following glycocalyx degradation, the *Bb* adhesion rate decreased. As mentioned above, previous studies have shown that removal of heparan sulfate and sialic acid residues decreases *Bb* adhesion, which we also observed (Figure 3a).^[^ ^70^, ^71^, ^72^, ^73^, ^74^ ^]^ However, despite the decrease in *Bb* adhesion, the extravasation rate did not significantly change (Figure 3b,c), implying that the glycocalyx does not play a role in *Bb* extravasation. This also suggests that adhesion may not be a necessary precursor to extravasation, and that some *Bb* may be able to directly transmigrate across the endothelium. Interestingly, when we assessed immune cell adhesion following glycocalyx degradation, we observed a significant increase in adhesion of THP‐1s and HL‐60s (Figure S8e,f, Supporting Information). One explanation for this differing response compared to *Bb* adhesion is that the glycocalyx acts as an obstacle for leukocytes to interact with adhesion molecules such as ICAM‐1 expressed on the surface of endothelial cells.^[^ ^78^, ^79^ ^]^ However, certain components of the glycocalyx (specifically heparan sulfate) act as targets for *Bb* adhesion, and degradation of these receptors decreases *Bb* adhesion. This highlights fundamental differences in the mechanism between *Bb* and leukocyte adhesion to endothelial cells.

Conversely, TNF‐α treatment to induce chronic inflammation in iEC microvessels did not significantly increase *Bb* adhesion rate compared to baseline conditions (Figure 4a). However, the rate and percentage of adhered *Bb* that extravasated both increased significantly during the third hour of perfusion (Figure 4b,c). This indicates that the increase in extravasation is a result of increased probability of extravasation of an adherent *Bb* rather than an increase in the number of adhered *Bb*.

### 3.5 Indirect Cytotoxicity Induced by *Borrelia burgdorferi*

EC activation can occur in response to inflammation or infection, leading to loss of barrier function and entry of blood components, immune cells, and pathogens into the brain, all of which can result in neuroinflammation and neurotoxicity.^[^ ^80^, ^81^, ^82^, ^83^ ^]^ However, we found that *Bb* alone, even at the high concentrations used here, did not result in EC activation and upregulation of ICAM‐1 expression (Figure 5e,f) or increased recruitment of immune cells (Figure 6c,d) in both iEC and iBMEC microvessels, similar to IVM studies.^[^ ^15^, ^16^ ^]^ This is likely due to the relatively short amount of time ECs were exposed to *Bb* (3 h), while peak upregulation of ICAM‐1 and neutrophil recruitment typically occur eight to twelve hours post infection.^[^ ^84^, ^85^ ^]^ This is consistent with the observation that symptoms associated with neuroborreliosis do not arise during the sub‐acute phase of infection.

Our results show that extravasation of *Bb* across the healthy BBB is a very rare event. If extravasation events occur by paracellular transport, these results suggest that the tight junctions formed between BMECs are sufficient to prevent *Bb* entry into the brain. This is supported by the observation that the permeability of iBMEC microvessels to 10 kDa dextran, which has a hydrodynamic radius of 4 nm and is much smaller than the approximate radius of *Bb* (165 nm), is extremely low.^[^ ^86^ ^]^ Furthermore, transient *Bb* adhesion alone or in conjunction with perfusion with inflammatory cytokines did not result in local or global loss of barrier function that could enable *Bb* extravasation through defects in the endothelial monolayer caused by downregulation of tight junctions in iBMECs (Figure 6 h; Figure S9h, Supporting Information). Together, these results suggest that the pathogenesis of neuroborreliosis via direct cytotoxicity is unlikely. Previous studies have shown that prolonged *Bb* infection can cause inflammation and increased production of CXCL13 in the cerebrospinal fluid (CSF), leading to lymphocytic pleocytosis.^[^ ^11^, ^14^, ^87^, ^88^, ^89^ ^]^ Reduction of inflammation leads to a decrease in focal neurodegeneration, suggesting indirect cytotoxicity as a possible model for the pathogenesis of neuroborreliosis.^[^ ^89^ ^]^ Although neuroinflammation caused by *Bb* infection is sufficient to cause neurological symptoms, it is unable to induce *Bb* extravasation across the BBB.

However, *Bb* perfusion does result in a significant increase in the global permeability of 2 MDa dextran in iEC microvessels after 3 h (Figure 6g). The absence of focal leaks suggests it is unlikely that the reduced barrier function is due to disruptions in cell‐cell junctions induced by extravasating *Bb*, and may be an alternative marker for the beginning of endothelial activation. As such, it is possible that circulating *Bb* are eliciting other changes to the endothelium, such as production of inflammatory cytokines.

## 4 Conclusion

**Figure 7:**
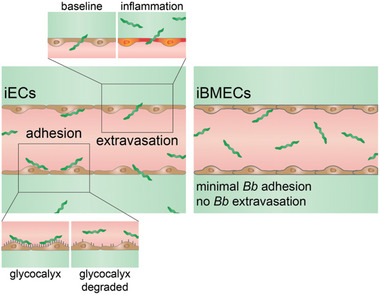
*Borrelia burgdorferi* (*Bb*) perfusion through tissue‐engineered microvessels. *Bb* adhere to and extravasate from iEC microvessels much more frequently than iBMEC microvessels. This implies that *Bb* crossing the BBB in humans is an extremely unlikely event, and that the pathogenesis of neuroborreliosis is through alternative mechanisms such as indirect cytotoxicity. *Bb* adhesion to endothelial cells is significantly decreased when the glycocalyx is degraded, while *Bb* extravasation into the surrounding ECM can be promoted via inflammation. *Bb* adhere to and extravasate from iEC microvessels much more frequently than iBMEC microvessels. This implies that *Bb* crossing the BBB in humans is an extremely unlikely event, and that the pathogenesis of neuroborreliosis is through alternative mechanisms such as indirect cytotoxicity. *Bb* adhesion to endothelial cells is significantly decreased when the glycocalyx is degraded, while *Bb* extravasation into the surrounding ECM can be promoted via inflammation.

In this study, we used a 3D tissue‐engineered microvessel to model *Bb* adhesion and extravasation in non‐tissue‐specific (iEC) and brain‐specific (iBMEC) microvessels (**Figure** **7**). Through real‐time imaging of *Bb*‐microvessel interactions under flow, we found that *Bb* adhesion and extravasation across the BBB is extremely rare, supporting observations in animal models and human autopsy data that *Bb* are rarely found in the brain parenchyma. However, *Bb* readily adhered and extravasated from iEC microvessels. *Bb* extravasation from iECs occurred via two steps: 1) endothelial adhesion, which involves the glycocalyx, and (2) transmigration into the ECM, which can be modulated via chronic inflammation. These findings highlight the advantages of using a reductive in vitro model to study *Bb* dissemination, and its capabilities for infectious disease research.^[^ ^90^, ^91^, ^92^ ^]^ To further improve the physiological relevance of our model, next steps include incorporating additional supporting cell types (e.g., pericytes, circulating immune cells) or using neurotropic *Borrelia* strains and species (e.g., *Bb* strain N40, *B. garinii*).

## 5 Experimental Section

### Fabrication of Microvessel Model

Fabrication of microvessels was done according to previously published protocols.^[^ ^61^, ^93^ ^]^ In short, cells were seeded into a 150 µm diameter channel in a 7 mg mL^−1^ type I collagen matrix (Corning, 354 249). iEC and iBMEC microvessels were formed by perfusing one to two microliters of cell solution at a density of 3 × 10^7^ or 8 × 10^7^ cells mL^−1^ respectively. After cells adhered, microvessels were maintained for 2 days at 5% CO_2_ and 37 °C to form a confluent monolayer. Further details of the protocol are described in the supplemental methods.

### *Borrelia burgdorferi* Perfusion

Microvessels were conditioned in co‐culture media at 37 °C for 24 h prior to perfusion with *Bb*. GFP‐labeled *Bb* (B31‐A3 GFP strain) was kindly provided by Dr. Utpal Pal (University of Maryland). Co‐culture medium was made by mixing in a one‐to‐one ratio BSK‐H with either EGM‐2 for iEC microvessels or iBMEC maintenance media for iBMEC microvessels. *Bb* were also conditioned in co‐culture media for 24 h at 37 °C. Perfusion was done with a syringe pump (Harvard Apparatus, 70–3010) at the indicated shear rate for each experiment for 3 h. During each hour of perfusion, to quantify interaction frequency, a 30‐to‐45‐min time‐lapse at an imaging rate of one frame per 15 s was acquired. After each hour of perfusion, adhered *Bb* was manually counted. After perfusion with *Bb* or vehicle (co‐culture media), functional assays were immediately performed.

### Glycocalyx Removal

Glycocalyx removal was done by perfusing a mix of 50 mU mL^−1^ of heparinase III (Millipore Sigma, H3917) and 20 mU mL^−1^ sialidase (Millipore Sigma, 10269611001) diluted in cell culture medium immediately following microvessel seeding. After maturation for 24 h, microvessels were switched to co‐culture media supplemented with the same concentration of heparinase III and sialidase for 24 h leading up to *Bb* perfusion.

### TNF‐α Treatment

Microvessels were perfused with 5 ng mL^−1^ of human TNF‐α recombinant protein (R&D Systems, 210‐TA) diluted in cell culture medium for 12 h prior to use for experiments.

### Permeability

Permeability assays were conducted one hour prior and immediately following perfusion with *Bb* or vehicle (co‐culture media). iEC and iBMEC microvessels were perfused with 2 µM fluorescein‐conjugated 2 MDa dextran (Thermo Fisher Scientific, cat. No. D7137) and 2 µM Alexa Fluor 647‐conjugated 10 kDa dextran (Thermo Fisher Scientific, cat. No. D22914) respectively in PBS. Phase and fluorescence images were captured at 10x magnification in a live cell chamber at 37 °C. Images were captured every 2 min for 10 min before and 30 min after perfusion of the dye solution. Permeability was then calculated using P = (r/2)(1/ΔI)(dI/dt) where r is the microvessel radius, ΔI is the increase in the lumen fluorescence intensity following injection of dye solution, and dI/dt is the rate of fluorescence intensity increase in the ECM.

### Immune Cell Adhesion

Immune cell adhesion assays were conducted immediately following permeability assays. THP‐1 cells (ATCC, TIB‐202) were stained with CellTracker deep red (Thermo Fisher Scientific, C34565) and HL‐60 cells (ATCC CCL‐240) were stained with Calcein‐AM (Thermo Fisher Scientific, C1430) for 20 min according to product protocols. Immune cells were then perfused through each microvessel for 10 min and non‐adherent cells were washed by perfusing co‐culture media for 30 min. Adherent THP‐1 and HL‐60s were manually counted in the fluorescence channel for each microvessel and normalized by microvessel length.

### Immunocytochemistry and Image Analysis

Immunocytochemistry was performed following permeability assays done after *Bb* or vehicle (co‐culture media) perfusion. Details of the protocol are described in the supplemental methods. Confocal z‐stacks (1 µm thickness) were acquired at 40x magnification on a spinning disk confocal microscope system (Nikon Yokogawa X1) and illumination was provided by a LUNF laser fixture (Nikon). To quantify the expression level of ICAM‐1 for each individual cell, a maximum intensity projection of 50 slices was created on the polar side of the microvessel. Boundaries for each individual cell were then demarcated manually using the ZO‐1 GFP fluorescence signal in Image J. The mean fluorescence intensity was then recorded. Reported data was normalized to the average fluorescence intensity of the DAPI nuclear stain of the projection.

### Statistics

All experimental values here are reported as mean with error bars denoting standard deviation (S.D.). Student's unpaired t‐test (two‐tailed with unequal variance) was used for comparison of two groups, while analysis of variance (ANOVA) was used for comparison of three or more groups. For comparison of adhesion and extravasation for each hour of perfusion, a repeated measures two‐way ANOVA was used. Statistically significant differences were defined as *p* < 0.05.

## Conflict of Interest

The authors declare no conflict of interest.

## Author Contributions

P.S. and L.W. conceived the original idea. L.W. and P.S. wrote the manuscript. L.W., Z.X., A.S., and B.M. contributed to the data acquisition and interpretation. All authors reviewed and edited the manuscript. P.S. supervised all work.

## Supporting information



Supporting Information

Supplemental Movie 1

Supplemental Movie 2

Supplemental Movie 3

Supplemental Movie 4

Supplemental Movie 5

Supplemental Movie 6

Supplemental Movie 7

Supplemental Movie 8

## Data Availability

The data that support the findings of this study are available from the corresponding author upon reasonable request.
