# Supplementary material for: Extravasation of Borrelia burgdorferi Across the Blood–Brain Barrier is an Extremely Rare Event
Source: Adv Sci (Weinh). 2025 Mar 12;12(17):2413199. doi: 10.1002/advs.202413199 (PMC12061299; doi:10.1002/advs.202413199)
Supplement: Supplementary file 1 — Supporting Information [file ADVS-12-2413199-s001.pdf]

ADVANCED SCIENCE
Open Access

Supporting Information

for *Adv. Sci.*, DOI 10.1002/advs.202413199

Extravasation of *Borrelia burgdorferi* Across the Blood–Brain Barrier is an Extremely Rare Event

*Linus Wang, Zikai Xia, Anjan Singh, Bhavna Murarka, Nicole Baumgarth, John N. Aucott and Peter C. Searson**

Supporting Information

Extravasation of *Borrelia burgdorferi* across the Blood-Brain Barrier is an Extremely Rare Event

*Linus Wang,[1,2], Zikai Xia[3], Anjan Singh [1,2], Bhavna Murarka[4], Nicole Baumgarth[5], John N. Aucott[6], Peter C. Searson[1,2,3]**

[1] Department of Biomedical Engineering, Johns Hopkins University
[2] Institute for Nanobiotechnology, Johns Hopkins University
[3] Department of Materials Science and Engineering, Johns Hopkins University
[4] Molecular and Cellular Biology, Johns Hopkins University
[5] Department of Molecular Microbiology and Immunology, Lyme and Tickborne Diseases Research and Education Institute, Johns Hopkins University
[6] Lyme Disease Research Center, Division of Rheumatology, Department of Medicine, Johns Hopkins University School of Medicine

Supplemental Notes
Note S1. *Bb* conditioning in co-culture media.
Note S2. Microvessel conditioning in co-culture media.
Note S3. *Bb*-microvessel interaction type classification.

Supplemental Figures
Figure S1. Microvessel model platform.
Figure S2. *Bb* conditioning in co-culture media.
Figure S3. Motility analysis of *Bb* conditioned in co-culture media.
Figure S4. Microvessel conditioning in co-culture media.
Figure S5. Timeline of microvessel preparation and *Bb* perfusion experiments.
Figure S6. Classification of types of *Bb*-microvessel interactions.
Figure S7. *Bb* adhesion and extravasation frequency in iEC and iBMEC microvessels.
Figure S8. Glycocalyx degradation of microvessels.
Figure S9. TNF-α induced inflammation of microvessels.
Figure S10. ICAM-1 fluorescence intensity of endothelial cells with adherent *Bb*.

Supplemental Tables:
Table S1. qPCR Primers.
Table S2. Antibodies used in this study.

Supplemental movies
Movie S1. *Bb* perfusion in iEC microvessel on equatorial plane.
Movie S2. *Bb* perfusion in iEC microvessel on polar plane.
Movie S3. *Bb* perfusion in iBMEC microvessel on equatorial plane.
Movie S4. Rotating 3D isometric view of *Bb* perfusion in iEC microvessel.
Movie S5. Quantification of *Bb* extravasation in iEC microvessel.
Movie S6. *Bb* transiently adhered to iEC microvessel.
Movie S7. *Bb* extravasation out of iEC microvessel.
Movie S8. *Bb* with unknown outcome in iEC microvessel.

Supplemental Methods
Cell Culture
Fabrication of Tissue-Engineered Model

Imaging
Borrelia burgdorferi Perfusion
Glycocalyx Degradation
TNF-α Treatment
Permeability
Immune Cell Adhesion
Immunocytochemistry and Image Analysis
Borrelia burgdorferi proliferation and viability assay
Borrelia burgdorferi qPCR
Borrelia burgdorferi 2D adhesion assay
Borrelia burgdorferi motility assay
Statistics

Supplementary Notes

Note S1. *Bb* conditioning in co-culture media.

Bb undergo significant changes in phenotype and gene expression when they are in an infected tick or host, and are sensitive to change depending on their environment.[1, 2] As such, we cultured *Bb* in various co-culture medias to determine the optimal medium for *Bb* perfusion experiments. We cultured *Bb* in BSK-H media and endothelial cell media, as well as co-culture media composed of 3 to 1, 1 to 1, and 1 to 3 mixtures of BSK-H to endothelial cell media (EGM-2 for iECs and iBMEC maintenance media for iBMECs) over three days to assess their proliferation and viability. *Bb* cultured in BSK-H and co-culture medias with EGM-2 showed similar growth profiles over three days (**Figure S2a**). *Bb* cultured in co-culture iBMEC maintenance media or endothelial cell media only did not proliferate over three days (**Figure S2b**). We defined viability as *Bb* maintaining an elongated morphology, while *Bb* round bodies were non-viable (**Figure S2c**). *Bb* cultured in BSK-H only or co-culture media (iEC and iBMEC) remained viable over all three days, while *Bb* in only endothelial cell media quickly loss viability over two days (**Figure S2d, e**).

In addition to proliferation and viability, we assessed changes in gene expression. After conditioning *Bb* in each media condition for 24 hours, we performed RNA extraction and qPCR for 3 gene targets: *bbk32*, *ospC*, and *bb0323*. BBK32 is a fibronectin and glycosaminoglycan (GAG)-binding protein that plays an important role in *Bb*-endothelial adhesion and interaction, OspC is an important surface protein that aids *Bb* in avoiding immune detection and is upregulated in the mammalian host, and BB0323 is a membrane associated lipoprotein involved in immune persistence, growth kinetics, and morphology.[3, 4] *Bb* conditioned in co-culture media with EGM-2 exhibited comparable expression levels of key genes as previous *in vivo* studies (**Figure S2f-h**).[4-6] *Bb* conditioned in co-culture media with iBMEC maintenance media up to a 1:1 mixture with BSK also exhibited similar gene expression levels as *in vivo* studies (**Figure S2i-k**).

To determine changes in *Bb* functionality, we coated glass wells with fibronectin and performed a 2D adhesion assay. *Bb* conditioned in BSK, 3:1, and 1:1 mixtures of BSK and endothelial media exhibited elevated levels of adhesion to fibronectin coated wells compared to control wells treated with PBS (**Figure S2l, m**).

We also assessed any differences in *Bb* motility. After conditioning *Bb* in co-culture media (1:1 mixtures of BSK-H to EGM-2 or iBMEC maintenance media), we inoculated *Bb* into a 7 mg mL^{-1} collagen gel in a 96-well plate and imaged time-lapses using confocal microscopy at an imaging frequency of 1 frame s^{-1}. For each condition, the migration path of over 60 *Bb* were recorded, and the displacement, instantaneous speed, and net speed were measured (**Figure S3**). There was no significant difference in the migratory behavior or activity of *Bb* in each media condition. Through these assays, we elected to use a co-culture media comprised of equal parts BSK-H and endothelial cell media for conditioning *Bb* and perfusion experiments.

Note S2. Microvessel conditioning in co-culture media.

After confirming that co-culture media comprised of a 1 to 1 mixture of BSK-H to endothelial cell media was suitable for *Bb* conditioning, microvessel tolerance for co-culture media was assessed. Microvessels were first seeded in EGM-2 or iBMEC induction media and allowed to mature for 24 hours. Then, microvessels were transferred to co-culture media and maintained for 24 hours. To determine whether co-culture media induces endothelial activation, we immunostained microvessels for surface adhesion molecule ICAM-1 and measured fluorescence intensity through confocal imaging (**Figure S4a, b**).[7] There was no significant change in ICAM-1 expression following 24 hours of maintenance in co-culture media (**Figure S4c, d**). The fluorescence intensity of the endogenously expressed tight junction marker ZO-1 was also compared, and no significant differences were observed (**Figure S4e, f**). Visual

examination of maximum intensity projected images also showed intact junctions across all conditions and no defects or breaks. These results suggest that the media combinations do not influence tight junction formation. We also performed immune cell adhesion assays to measure functional changes in endothelial activation, and observed no changes in recruitment of THP-1s or HL-60s (**Figure S4g, h**). We confirmed barrier function integrity by measuring the permeability of fluorescently labeled 2 MDa or 10 kDa dextran for iEC or iBMEC microvessels respectively and found no significant difference (**Figure S4i, j**).

Note S3. *Bb*-microvessel interaction type classification.

To quantify *Bb*-microvessel interaction type and frequency during perfusion, time-lapse images were taken every 15 seconds over 30 to 45 minutes (**Figure S6**). The plane of focus was always at the equatorial plane to accurately confirm complete *Bb* transmigration during extravasation events. For an interaction to be included for analysis, a *Bb* must have remained in focus for at least 2 frames. As a result, all recorded interactions last a minimum of 30 seconds due to the imaging frequency. This is a much stricter criterion for counting interactions than other studies, which use the speed at which *Bb* travel across the endothelium.[8-12] In our 150 μm diameter microvessel, we can only image one plane every 15 seconds while simultaneously imaging multiple points along the length of the microvessel to record enough interactions for analysis. As such, interactions lasting less than 30 seconds were not captured, but we were still able to compare interaction type and frequency for different experimental conditions (vessel type, glycocalyx degradation, TNF-α etc.) in our study.

After initial adhesion, one of three types of interactions occurred (**Figure S6**). (1) Transient adhesion occurred if a *Bb* detached after adhering to the microvessel and is washed away in flow. (2) Extravasation occurred if a *Bb* transmigrated across the endothelium into the ECM, and is complete when the *Bb* is fully in the ECM and separated from the microvessel. (3) Unknown outcomes occurred when a *Bb* adhered to the microvessel during the time-lapse and remained adhered at the end of the imaging period (lasting 30 to 45 minutes). The *Bb* remained in focus, but the eventual outcome of the *Bb* was unknown because of the imaging setup. This upper limit is due to the need to quantify the adhesion and extravasation frequency of *Bb* along the microvessel after each hour of perfusion. As such, this sets an upper limit to the duration of interactions at 45 minutes or 2700 seconds, which is well above the mean duration of both transient and extravasation interactions. If the imaging period were to last indefinitely, we expect these Bb to eventually follow one of the two types of outcomes outlined above.

In addition, we measured the adhesion and extravasation frequency after each hour of perfusion. To quantify adhesion frequency, we counted the total number of adherent *Bb* across the microvessel (**Figure S7a**). To quantify extravasation frequency, we determined the number of *Bb* that came into focus in the surrounding ECM at the equatorial plane over a one minute time-lapse (**Figure S7b, Movie S5**).

Supplemental Figures

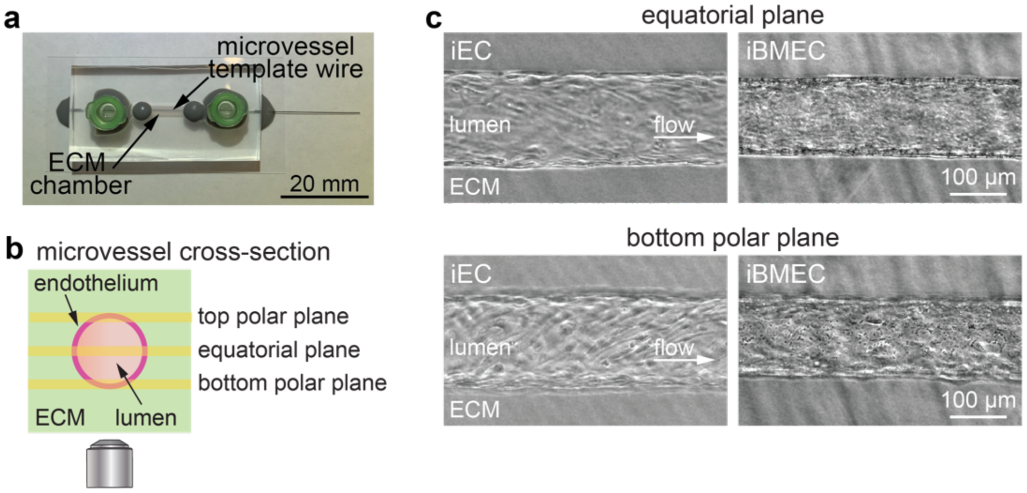

Figure S1. Microvessel model platform.
(a) Photograph of the microfluidic platform used for microvessel models. A 150 µm diameter nitinol rod is suspended in a collagen I gel and removed to template a channel for seeding endothelial cells to form microvessels.
(b) Schematic illustration of the microvessel cross-section and the different imaging planes.
(c) Representative phase contrast images of the equatorial and bottom polar planes of an induced endothelial cell (iEC) and induced brain microvascular endothelial cell (iBMEC) microvessel 48 h after seeding.

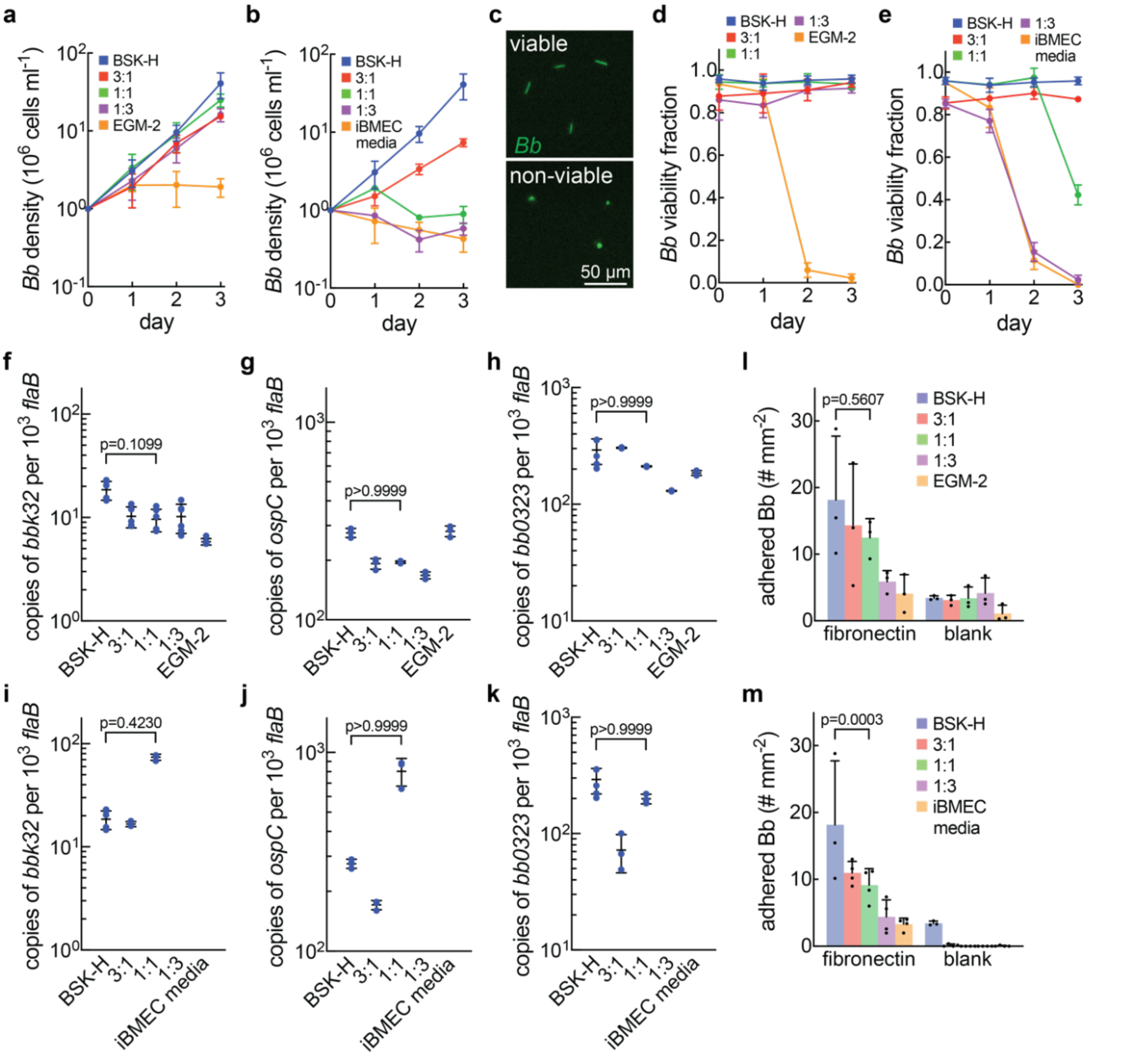

Figure S2. *Borrelia burgdorferi* (*Bb*) conditioning in co-culture media.
(a) *Bb* proliferation over three days of culture in co-culture media with EGM-2. n = 3 for each condition.
(b) *Bb* proliferation over three days of culture in co-culture media with iBMEC media. n = 3 for each condition.
(c) Representative fluorescence images of viable, elongated *Bb* and non-viable, rounded *Bb*.
(d) Fraction of viable *Bb* over three days of culture in co-culture media with EGM-2. Viability is determined by the morphology of the *Bb*, where elongated *Bb* are viable and rounded *Bb* are non-viable. n = 3 for each condition.
(e) Fraction of viable *Bb* over three days of culture in co-culture media with iBMEC media. Viability is determined by the morphology of the *Bb*, where is elongated *Bb* are viable and rounded *Bb* are non-viable. n = 3 for each condition.
(f) qPCR of *bbk32* expression relative to *flaB* for *Bb* after 24 hours of culture in co-culture media with EGM-2. n = 5 for each condition.
(g) qPCR of *ospC* expression relative to *flaB* for *Bb* after 24 hours of culture in co-culture media with EGM-2. n = 3 for each condition.
(h) qPCR of *bb0323* expression relative to *flaB* for *Bb* after 24 hours of culture in co-culture media with EGM-2. n = 3 for each condition.

(i) qPCR of *bbk32* expression relative to *flaB* for *Bb* after 24 hours of culture in co-culture media with iBMEC media. n = 3 for each condition.
(j) qPCR of *ospC* expression relative to *flaB* for *Bb* after 24 hours of culture in co-culture media with iBMEC media. n = 3 for each condition.
(k) qPCR of *bb0323* expression relative to *flaB* for *Bb* after 24 hours of culture in co-culture media with iBMEC media. n = 3 for each condition.
(l) 2D adhesion to 20 μg mL^{-1} fibronectin coated or blank (PBS treated) glass bottom wells after 24 hours of *Bb* culture in co-culture media with EGM-2. n = 3 for each condition.
(m) 2D adhesion to 20 μg mL^{-1} fibronectin coated or blank (PBS treated) glass bottom wells after 24 hours of *Bb* culture in co-culture iBMEC media. n = 3 for BSK-H, n = 4 for all other conditions.

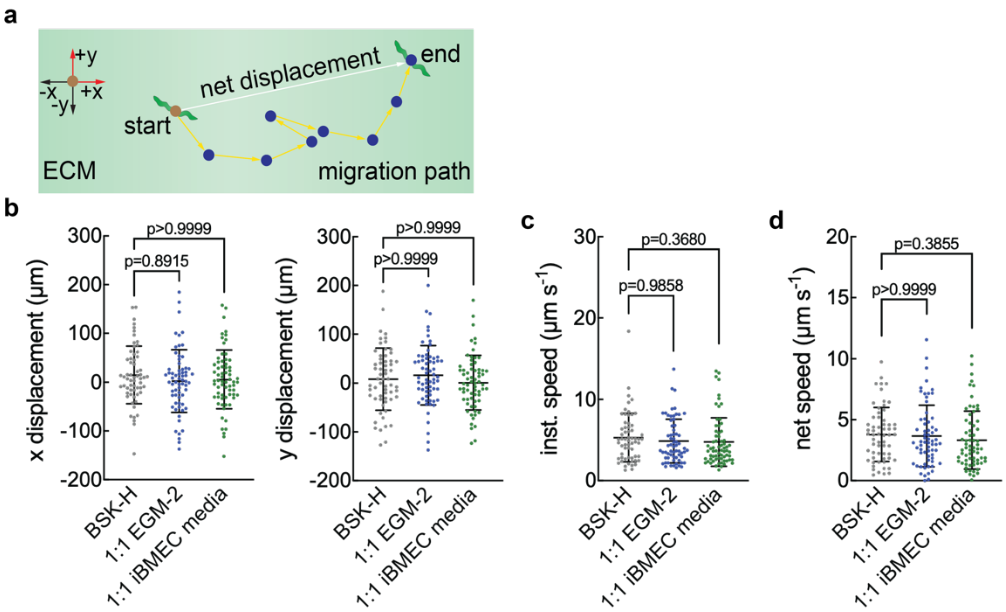

Figure S3. Motility analysis of *Borrelia burgdorferi* (*Bb*) conditioned in co-culture media.
(a) Schematic illustration of *Bb* migration in collagen I gel. Images were acquired every second over 5 min. Motility analysis was limited only to *Bb* present in the focal plane for at least 20 s.
(b) Net displacement of *Bb* after 24 h of conditioning in BSK-H or co-culture media. BSK-H n = 61. 1:1 EGM-2 co-culture media n = 65. 1:1 iBMEC co-culture media n = 67.
(c) Instantaneous speed of *Bb* after 24 h of conditioning in BSK-H or co-culture media. BSK-H n = 61. 1:1 EGM-2 co-culture media n = 65. 1:1 iBMEC co-culture media n = 67.
(d) Net speed of *Bb* after 24 h of conditioning in BSK-H or co-culture media. BSK-H n = 61. 1:1 EGM-2 co-culture media n = 65. 1:1 iBMEC co-culture media n = 67.

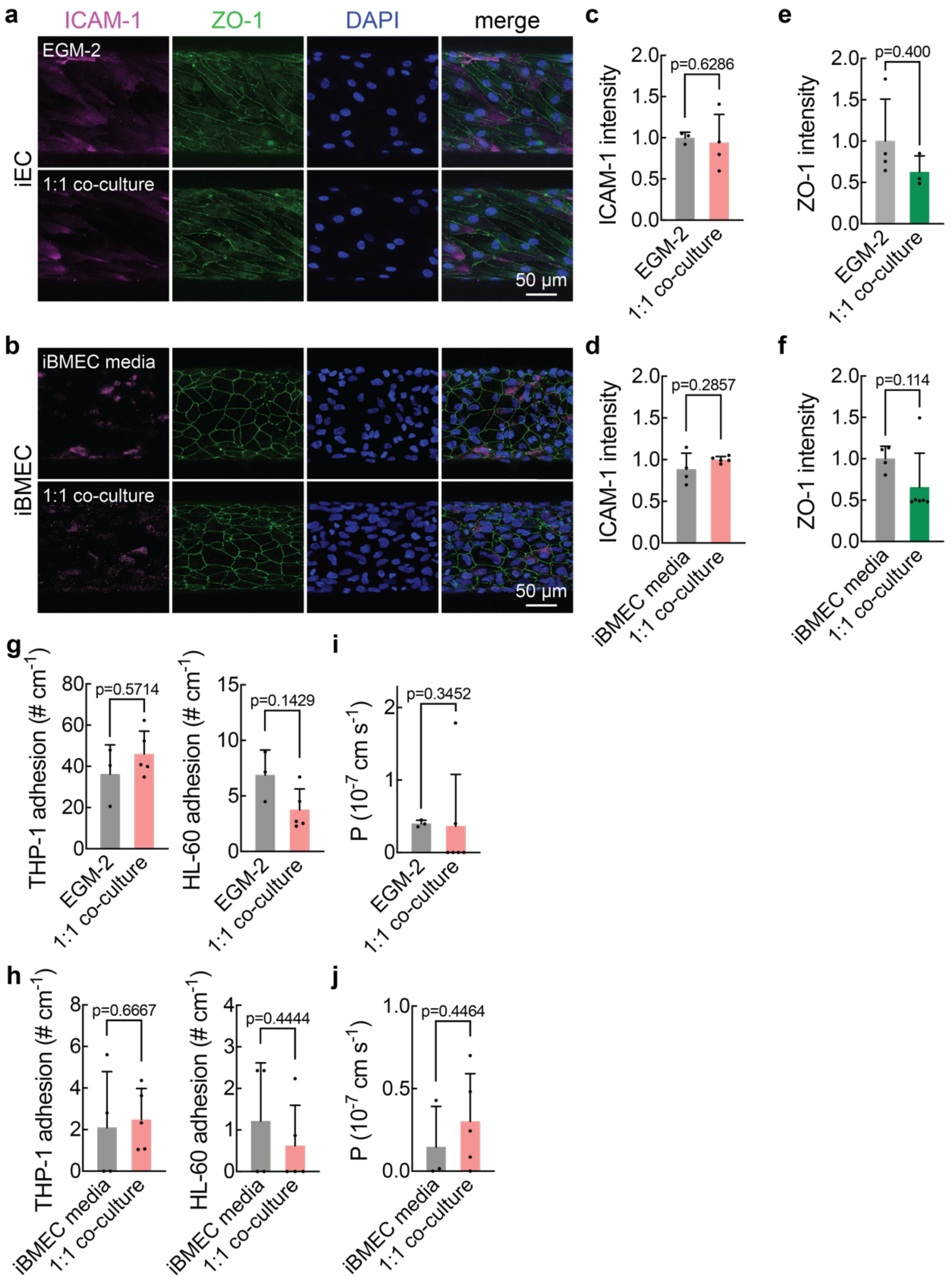

Figure S4. Microvessel conditioning in co-culture media.
(a) Representative maximum intensity projection confocal images of ICAM-1 (magenta) immunostained iEC microvessels with ZO-1 (green) and DAPI (blue) after perfusion with 1:1 co-culture media or EGM-2.
(b) Representative maximum intensity projection confocal images of ICAM-1 (magenta) immunostained iBMEC microvessels with ZO-1 (green) and DAPI (blue) after perfusion with 1:1 co-culture media or iBMEC maintenance media.
(c) Mean ICAM-1 fluorescence in iEC microvessels from maximum intensity projection confocal images over 50 µm after perfusion with 1:1 co-culture media or EGM-2. 1:1 co-culture media n = 4. EGM-2 n = 3.

(d) Mean ICAM-1 fluorescence in iBMEC microvessels from maximum intensity projection confocal images over 50 μm after perfusion with 1:1 co-culture media or iBMEC maintenance media. 1:1 co-culture media n = 5. iBMEC maintenance media n = 4.
(e) Mean ZO-1 fluorescence in iEC microvessels from maximum intensity projection confocal images over 50 μm after perfusion with 1:1 co-culture media or EGM-2. 1:1 co-culture media n = 4. EGM-2 n = 3.
(f) Mean ZO-1 fluorescence in iBMEC microvessels from maximum intensity projection confocal images over 50 μm after perfusion with 1:1 co-culture media or iBMEC maintenance media. 1:1 co-culture media n = 5. iBMEC maintenance media n = 4.
(g) THP-1 and HL-60 adhesion in iEC microvessels after perfusion with 1:1 co-culture media or EGM-2. 1:1 co-culture media n = 5. EGM-2 n = 3.
(h) THP-1 and HL-60 adhesion in iBMEC microvessels after perfusion with 1:1 co-culture media or iBMEC maintenance media. 1:1 co-culture media n = 5. iBMEC maintenance media n = 4.
(i) Permeability of 2 MDa dextran in iEC microvessels after perfusion with 1:1 co-culture media or EGM-2. 1:1 co-culture media n = 6. EGM-2 n = 3.
(j) Permeability of 10 kDa dextran in iBMEC microvessels after perfusion with 1:1 co-culture media or iBMEC maintenance media. 1:1 co-culture media n = 5. iBMEC maintenance media n = 3.

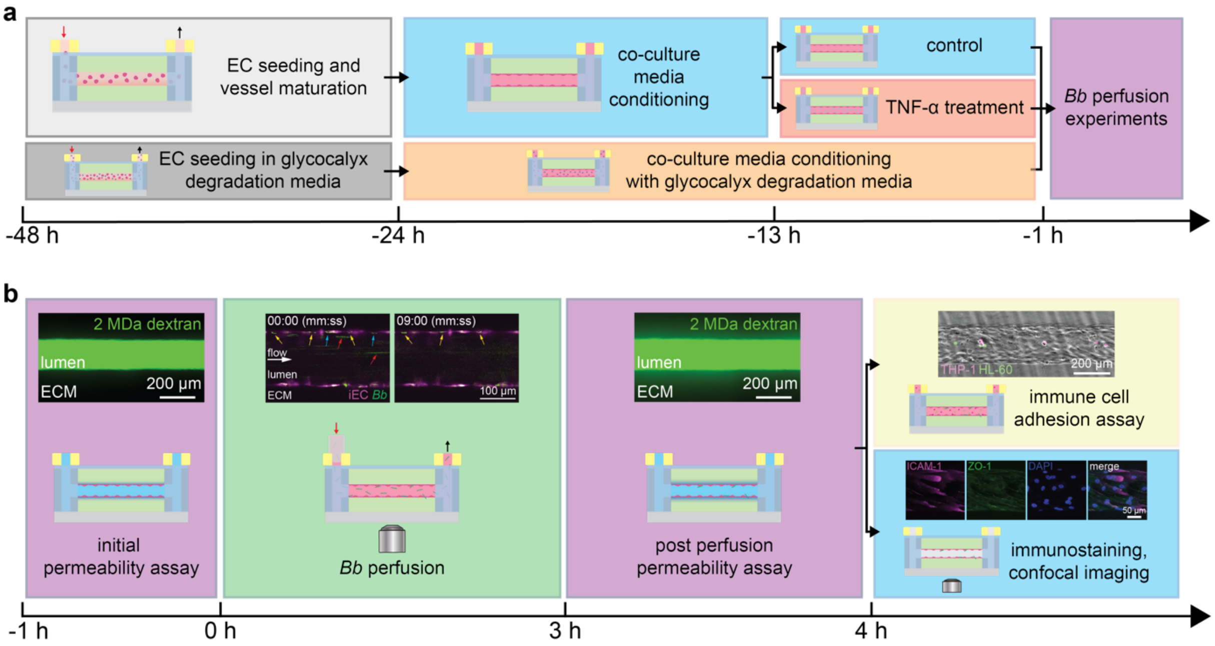

Figure S5. Timeline of microvessel preparation and *Borrelia burgdorferi* (*Bb*) perfusion experiments.

(a) Timeline of microvessel seeding and culture prior to usage in *Bb* perfusion experiments. Endothelial cells (ECs) were first seeded and allowed to mature into microvessels in EC medium. Microvessels undergoing glycocalyx degradation were treated with heparinase III and sialidases immediately following seeding in EC medium, and continued to be treated after switching to co-culture medium after 24 hours of maturation. In the other conditions, microvessels were conditioned in co-culture medium 24 hours after maturation, and then subjected to one of the two remaining experimental conditions: (1) control, where the microvessel remained in co-culture medium, or (2) TNF-α, where the microvessel is treated with 5 ng mL^{-1} of TNF-α suspended in co-culture medium for 12 hours.

(b) Timeline of *Bb* perfusion experiments and assays with representative images. An initial permeability assay was conducted to measure permeability and confirm the barrier integrity of the microvessel. *Bb* was then perfused for three hours. A post-perfusion permeability assay was conducted to measure the change in permeability. The microvessel was then used for immune cell adhesion assays or for immunostaining and confocal imaging.

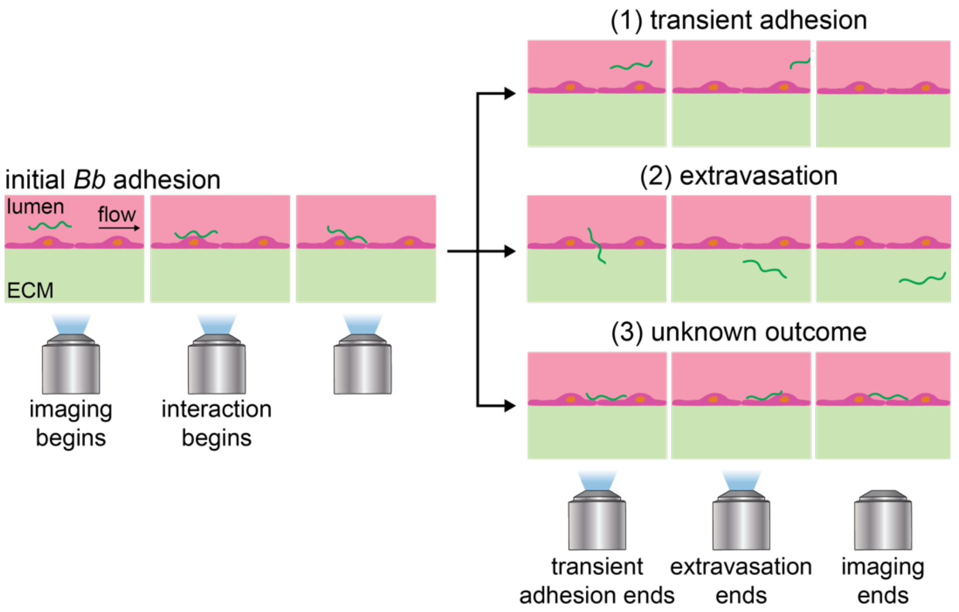

Figure S6. Classification of types of *Borrelia burgdorferi* (*Bb*)-microvessel interactions. Schematic illustration of the 3 different types of *Bb*-microvessel interactions observed in this study. Once the imaging period begins, *Bb* that remain in focus at the same location in the microvessel are counted as an interaction. After initial *Bb* adhesion, one of three different types of interactions may occur. (1) Transient adhesion, where the *Bb* detaches from the microvessel and is washed away in flow. (2) Extravasation, where the *Bb* transmigrates across the microvessel and enters the ECM. Extravasation is considered complete when the *Bb* fully dissociates from the endothelium. (3) Unknown outcome, where the *Bb* remains adhered at the end of the imaging period.

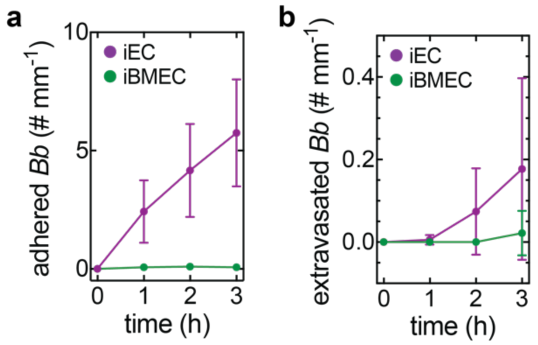

Figure S7. *Borrelia burgdorferi* (*Bb*) adhesion and extravasation frequency in induced endothelial cell (iEC) and induced brain microvascular endothelial cell (iBMEC) microvessels.
(a) *Bb* adhesion frequency normalized by microvessel length after each hour of perfusion in iEC or iBMEC microvessels. iEC n = 6. iBMEC n = 6.
(b) *Bb* extravasation frequency normalized by microvessel length after each hour of perfusion in iEC or iBMEC microvessels. Extravasation was quantified by taking images every minute at the equatorial plane and counting the number of *Bb* that came into focus. iEC n = 4. iBMEC n = 6.

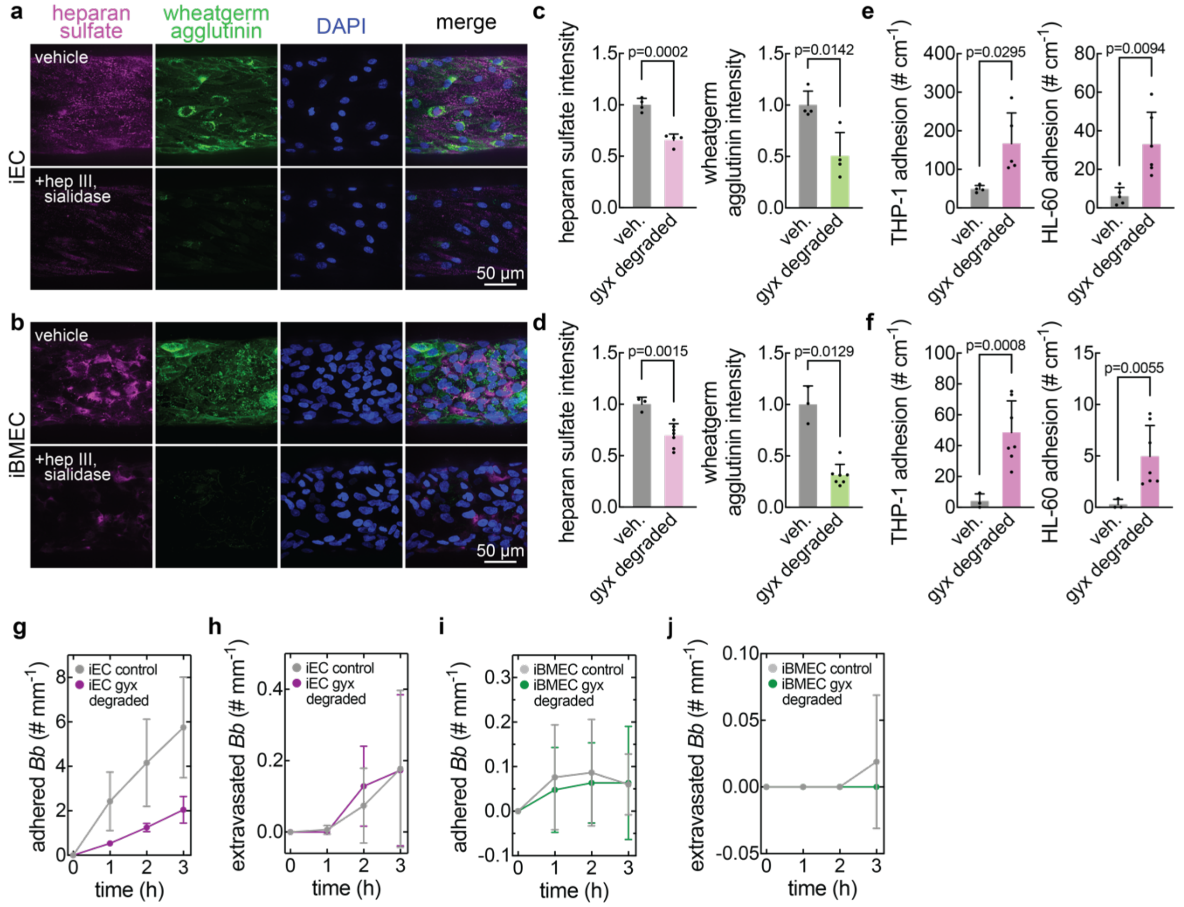

Figure S8. Glycocalyx degradation of microvessels.
(a) Representative maximum intensity projection confocal images of iEC microvessels treated with vehicle or heparinase III and sialidase. Microvessels were immunostained with heparan sulfate (magenta) and wheatgerm agglutinin (green).
(b) Representative maximum intensity projection confocal images of iBMEC microvessels treated with vehicle or heparinase III and sialidase. Microvessels were immunostained with heparan sulfate (magenta) and wheatgerm agglutinin (green).
(c) Mean heparan sulfate and wheatgerm agglutinin fluorescence in iEC microvessels from maximum intensity projection confocal images after treatment with heparinase III and sialidase or vehicle. Glycocalyx degraded n = 4. Vehicle n = 4.
(d) Mean heparan sulfate and wheatgerm agglutinin fluorescence in iBMEC microvessels from maximum intensity projection confocal images after treatment with heparinase III and sialidase or vehicle. Glycocalyx degraded n = 7. Vehicle n = 3.
(e) THP-1 and HL-60 adhesion in iEC microvessels after treatment with heparinase III and sialidase or vehicle. Glycocalyx degraded n = 5. Vehicle n = 5.
(f) THP-1 and HL-60 adhesion in iBMEC microvessels after treatment with heparinase III and sialidase or vehicle. Glycocalyx degraded n = 7. Vehicle n = 3.
(g) *Bb* adhesion frequency normalized by microvessel length after each hour of perfusion in glycocalyx degraded or control iEC microvessels. Glycocalyx degraded n = 5. Control n = 6.
(h) *Bb* extravasation frequency normalized by microvessel length after each hour of perfusion in glycocalyx degraded or control iEC microvessels. Glycocalyx degraded n = 5. Control n = 6.
(i) *Bb* adhesion frequency normalized by microvessel length after each hour of perfusion in glycocalyx degraded or control iBMEC microvessels. Glycocalyx degraded n = 4. Control n = 6.

(j) *Bb* extravasation frequency normalized by microvessel length after each hour of perfusion in glycocalyx degraded or control iBMEC microvessels. Glycocalyx degraded n = 4. Control n = 6.

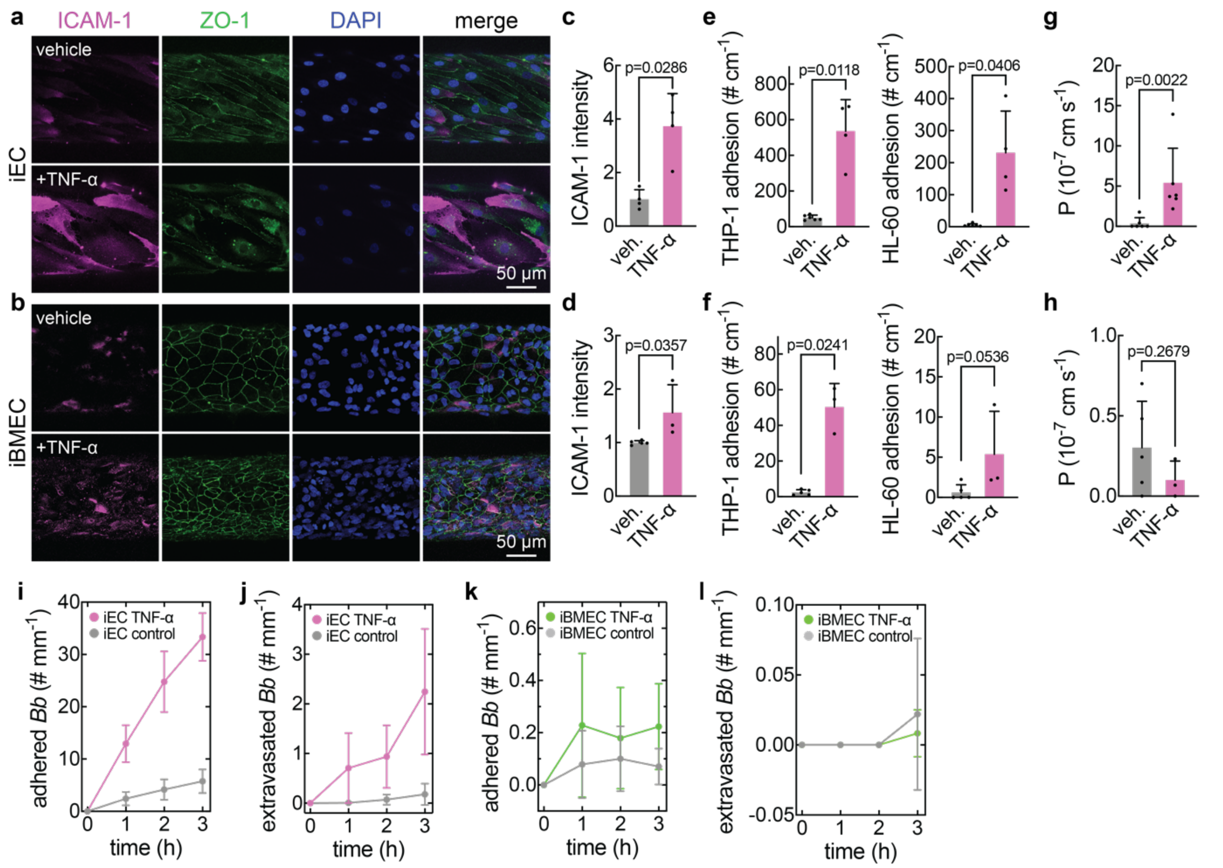

Figure S9. TNF-α induced inflammation of microvessels.
(a) Representative maximum intensity projection confocal images of ICAM-1 (magenta) immunostained iEC microvessels with ZO-1 (green) and DAPI (blue) after 12 hours of treatment with 5 ng mL^{-1} TNF-α or vehicle.
(b) Representative maximum intensity projection confocal images of ICAM-1 (magenta) immunostained iBMEC microvessels with ZO-1 (green) and DAPI (blue) after 12 hours of treatment with 5 ng mL^{-1} TNF-α or vehicle.
(c) Mean ICAM-1 fluorescence in iEC microvessels from maximum intensity projection confocal images over 50 µm after 12 hours of treatment with 5 ng mL^{-1} TNF-α or vehicle. TNF-α n = 4. Vehicle n = 4.
(d) Mean ICAM-1 fluorescence in iBMEC microvessels from maximum intensity projection confocal images over 50 µm after 12 hours of treatment with 5 ng mL^{-1} TNF-α or vehicle. TNF-α n = 3. Vehicle n = 5.
(e) THP-1 and HL-60 adhesion in iEC microvessels after 12 hours of treatment with 5 ng mL^{-1} TNF-α or vehicle. TNF-α n = 4. Vehicle n = 6.
(f) THP-1 and HL-60 adhesion in iBMEC microvessels after 12 hours of treatment with 5 ng mL^{-1} TNF-α or vehicle. TNF-α n = 3. Vehicle n = 5.
(g) Permeability of 2 MDa dextran in iEC microvessels after 12 hours of treatment with 5 ng mL^{-1} TNF-α or vehicle. TNF-α n = 6. Vehicle n = 6.
(h) Permeability of 10 kDa dextran in iBMEC microvessels after 12 hours of treatment with 5 ng mL^{-1} TNF-α or vehicle. TNF-α n = 3. Vehicle n = 5.
(i) *Bb* adhesion frequency normalized by microvessel length after each hour of perfusion in TNF-α treated or control iEC microvessels. TNF-α n = 4. Control n = 6.
(j) *Bb* extravasation frequency normalized by microvessel length after each hour of perfusion in TNF-α treated or control iEC microvessels. TNF-α n = 4. Control n = 6.

(k) *Bb* adhesion frequency normalized by microvessel length after each hour of perfusion in TNF-α treated or control iBMEC microvessels. TNF-α n = 4. Control n = 6.
(l) *Bb* extravasation frequency normalized by microvessel length after each hour of perfusion in TNF-α treated or control iBMEC microvessels. TNF-α n = 4. Control n = 6.

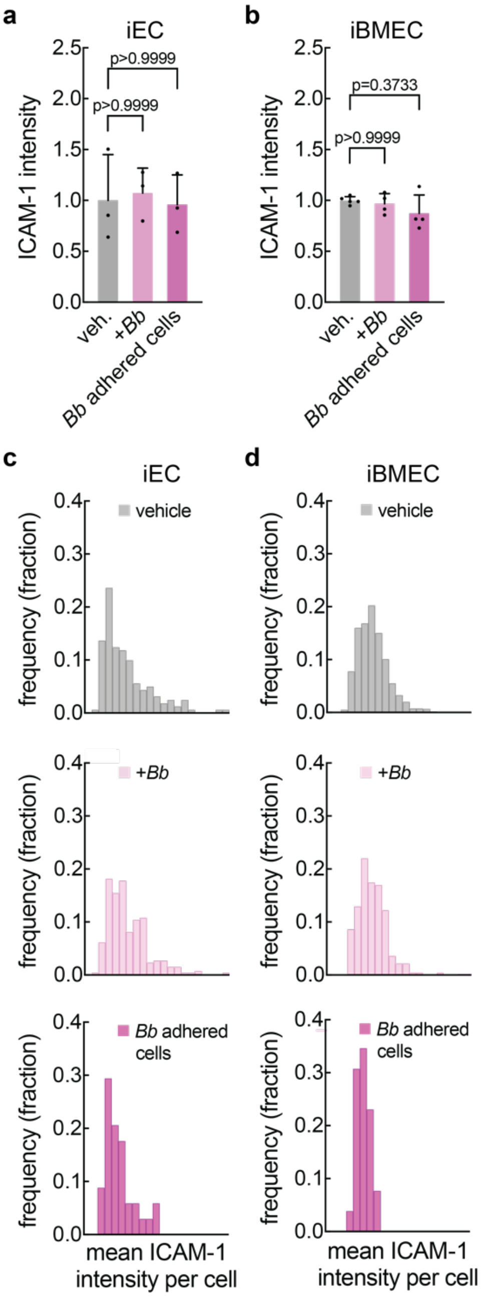

Figure S10. Analysis of ICAM-1 fluorescence intensity of endothelial cells with adherent *Borrelia burgdorferi* (*Bb*). For representative immunofluorescence images see Fig. 5.

(a) Mean ICAM-1 fluorescence in iEC microvessels from maximum intensity projection confocal images over 50 µm after three hours of perfusion with *Bb* or vehicle. +*Bb* n = 3. *Bb* adhered cells n = 3. Vehicle n = 3.

(b) Mean ICAM-1 fluorescence in iBMEC microvessels from maximum intensity projection confocal images over 50 µm after three hours of perfusion with *Bb* or vehicle. +*Bb* n = 4. *Bb* adhered cells n = 4. Vehicle n = 6.

(c) Histogram of individual iEC cell ICAM-1 fluorescence after three hours of perfusion with *Bb* or vehicle. +*Bb* n = 161 cells across 3 independent microvessels. *Bb* adhered cells n = 34 cells across 3 independent microvessels. Vehicle n = 259 individual cells across 3 independent microvessels.

(d) Histogram of individual iBMEC cell ICAM-1 fluorescence after three hours of perfusion with *Bb* or vehicle. +*Bb* n = 418 cells across 4 independent microvessels. *Bb* adhered cells n = 26 cells across 4 independent microvessels. Vehicle n = 518 individual cells across 6 independent microvessels.

Supplemental Tables

Table S1. qPCR Primers.

Gene	Sequence (5' – 3')	Reference
flaB (forward)	TTG CTG ATC AAG CTC AAT ATA ACC A	Bista, 2020[13]
flaB (reverse)	TTG AGA CCC TGA AAG TGA TGC	
bbk32 (forward)	CAA CAA AGC TAA CCC AAA TGT AT	Li, 2006[5]
bbk32 (reverse)	CTT TTG TAA ACT TTG CAG CTT CT	
ospC (forward)	GCTGATGAGTCTGTTAAAGGG	Yang, 1999[14]
ospC (reverse)	GCATCTCTTTAGCTGCTTTTG	
bb0323 (forward)	ATATGGATCCCGCTGGAAAT	Kariu, 2015[15]
bb0323 (reverse)	AGCCGCTTCAAGTGCTTTTA	

Table S2. Antibodies used in this study.

Antibody	Vendor	Species	Cat. No	Dilution
ICAM-1	Abcam	Mouse	ab2213	1:40
Heparan Sulfate	USBiological Life Sciences	Mouse	H1890	1:40
Wheat Germ Agglutinin Alexa Fluor™ 488	Invitrogen	Wheat Germ	W11261	1:200
Alexa Fluor™ 647	Invitrogen	Goat	A-21235	1:200

Supplemental movies

Movie S1. Bb perfusion in iEC microvessel on equatorial plane.
Bb at a density of 5 x 10^6 cells mL^{-1} are perfused at a shear rate of 1 dyne cm^{-2} in an iEC microvessel stained with CellTracker deep red. The plane of focus is at the equatorial plane of the microvessel, and *Bb* perfusing through the lumen can be seen to interact with the sides of the microvessel.

Movie S2. Bb perfusion in iEC microvessel on polar plane.
Bb perfusion in the same microvessel under the same conditions as that of movie S1, but the plane of focus is at the polar plane.

Movie S3. Bb perfusion in iBMEC microvessel on equatorial plane.
Bb at a density of 5 x 10^6 cells mL^{-1} are perfused at a shear rate of 1 dyne cm^{-2} in an iBMEC microvessel stained with CellTracker deep red. The plane of focus is at the equatorial plane of the microvessel.

Movie S4. Rotating 3D isometric view of Bb perfusion in iEC microvessel.
3D reconstruction of an iEC microvessel stained with CellTracker deep red and perfused with *Bb*.

Movie S5. Quantification of Bb extravasation in iEC microvessel.
Extravasated *Bb* can be seen migrating in the surrounding ECM. The number of *Bb* that come into the plane of focus at the equatorial plane over 1 minute is counted to quantify *Bb* extravasation. In this example, there are three extravasated *Bb* in the ECM.

Movie S6. Bb transiently adhered to iEC microvessel.
Bb undergoes initial adhesion at approximately 7 seconds on the lumen (top) of the iEC microvessel. The *Bb* drags across the cell briefly before detaching at approximately 1 minute 49 seconds.

Movie S7. Bb extravasation out of iEC microvessel.
Bb undergoes initial adhesion at approximately 1 second on the lumen (top) of the iEC microvessel. The *Bb* transmigrates across the microvessel over several minutes, and begins to enter the ECM at approximately 8 minutes. The *Bb* fully extravasates at approximately 8 minutes 45 seconds and begins migrating through the ECM.

Movie S8. Bb with unknown outcome in iEC microvessel.
A *Bb* is initially adhered, which is not included in analysis. At approximately 5 minutes, a second *Bb* adheres to the microvessel. The *Bb* remains adherent and in focus, but the imaging period ends at 20 minutes, and the outcome of the *Bb* is not known.

Supplemental Methods

Cell Culture

Tissue-engineered microvessels were generated using isogenic induced pluripotent stem cell (iPSC)-derived endothelial cells (iECs) and brain microvascular endothelial-like cells (iBMECs). Differentiations were done from an iPSC cell line from a healthy donor (Allen Cell Collection, WTC parent) that expresses fluorescently labeled zona occludens-1 (ZO-1).

iECs were differentiated using *ETV2* modRNA using published protocols.[16] iECs were cultured in Endothelial Cell Growth Medium-2 (EGM-2; Lonza, CC-3202) supplemented with 10 μM SB431542 (Selleckchem, S1067). Note that the hydrocortisone provided in the supplementary kit (Lonza, CC-4147) was not included, and the GA-1000 was replaced with gentamicin at a final concentration of 30 μg mL^{-1}, as the *Bb* strain used in the study is gentamicin resistant.

iBMECs were differentiated using published protocols.[17] iBMECs were plated onto 6-well plates coated with 50 μg mL^{-1} human placental collagen IV (Sigma-Aldrich, C5533) and 25 μg mL^{-1} fibronectin from human plasma (Sigma-Aldrich, F2006) for 1 hour at 37 ˚C prior to seeding. During seeding and the following 24 hours, iBMECs were cultured in induction medium composed of human endothelial cell serum-free medium (HESFM; Thermo Fisher Scientific, 11111044) supplemented with 1% human serum from platelet-poor plasma (Sigma-Aldrich, P2918), 1% penicillin-streptomycin (Thermo Fisher Scientific, 15140122), 2 ng mL^{-1} human recombinant basic fibroblast growth factor (Thermo Fisher Scientific, 233FB025CF), and 10 μM all-trans retinoic acid (Sigma-Aldrich, R2625).

The *Bb* (B31-3 GFP strain) was cultured in BSK-H medium at 37 ˚C with 5% CO_2. The *Bb* used throughout the study were kept under passage 6 to avoid loss of endogenous plasmids caused during passaging.

Fabrication of Tissue-Engineered Model

Fabrication of the microfluidic platform for generating microvessel models was based on our previous work.[18, 19] To model the extracellular matrix, 7 mg mL^{-1} neutralized collagen I solution (Corning, 354249) was injected into each microfluidic device at room temperature and then gelled for 10 to 15 minutes at 37 ˚C. Then, the template rod was removed to create a 150 μm diameter channel.

For iEC microvessels, prior to seeding, devices were perfused with EGM-2 overnight at 37 ˚C. iECs were then detached using 0.25% Trypsin-EDTA (Thermo Fisher Scientific, 25200056) and resuspended at a concentration of 3 x 10^7 cells mL^{-1}. Devices were then perfused with 1-2 μL of the high concentration cell solution and allowed to adhere under static conditions before washing out nonadherent cells with EGM-2. Microvessels were then perfused with EGM-2 under an average shear stress of 4 dyne cm^{-2} for 24 hours at 37 ˚C.

For iBMEC microvessels, prior to seeding, devices were coated with 50 μg mL^{-1} human placental collagen IV and 25 μg mL^{-1} fibronectin from human plasma diluted in iBMEC induction media overnight at 37 ˚C. iBMECs were then detached using Accutase (Thermo Fisher Scientific, A1110501) and resuspended at a concentration of 8 x 10^7 cells mL^{-1}. Devices were then perfused with 1-2 μL of the high concentration cell solution and allowed to adhere under static conditions before washing out nonadherent cells with iBMEC induction media. Microvessels were then perfused with iBMEC induction media under an average shear stress of 4 dyne cm^{-2} for 24 hours at 37 ˚C.

Imaging

Phase contrast, permeability, immune cell adhesion, and *Bb* counting images were acquired on an inverted microscope (Nikon Eclipse TiE) at 10x magnification. Epifluorescence

illumination was controlled by X-Cite 120LED Boost (Excelitas Technologies). Whole microvessel images were assembled from 10 images of approximately 700 μm segments along in length.

Time-lapse fluorescence images were acquired at 20x magnification on a spinning disk confocal microscope system (Nikon Yokogawa X1) and illumination was provided by a LUNF laser fixture (Nikon). For interaction frequency and type quantification, the image rate was 1 frame per 15 seconds over 30 to 45 minutes at the equatorial plane. For adhesion quantification, the focus was manually adjusted from the bottom to the top polar plane, and adhered *Bb* were counted. For extravasation quantification, the image rate was 1 frame per second over 1 minute at the equatorial plane. All imaging was done in a live cell chamber at 37 ˚C. To visualize the endothelium, in some experiments, endothelial cells were stained with CellTracker Deep Red (Thermo Fisher Scientific, C34565). iECs were stained in a T-25 flask according to product protocols 1 day prior to seeding in the device. iBMECs were stained in the device according to product protocols 1 day after seeding.

Borrelia burgdorferi Perfusion

Microvessels were conditioned in co-culture media at 37 ˚C for 24 hours prior to perfusion with *Bb*. Co-culture medium was made by mixing in a 1 to 1 ratio BSK-H with either EGM-2 for iEC microvessels or iBMEC maintenance media, which is composed of HESFM supplemented with 5% fetal bovine serum (Sigma-Aldrich, F4315) and 30 μg mL^{-1} gentamicin. *Bb* were collected by centrifugation at 6000 x g for 10 minutes, resuspended at a density of approximately 1 x 10^7 cells mL^{-1} in the co-culture medium, and incubated at 37 ˚C for 24 hours.

Before perfusing with *Bb* or vehicle (co-culture media), an initial permeability assay was performed on each microvessel to confirm the formation of a confluent monolayer without any focal leaks. The protocol for permeability assays is detailed in a following section. Perfusion was done with a syringe pump (Harvard Apparatus, 70-3010) at the indicated shear rate for each experiment for three hours.

During each hour of perfusion, to quantify interaction frequency, a 30-to-45-minute time-lapse at an imaging rate of 1 frame per 15 seconds was acquired. This was done at 5 to 7 locations equidistantly distributed along the microvessel, where the field of view for each location was approximately 400 μm in length. *Bb*-microvessel interactions were included in analysis if the *Bb* remained in focus for at least two frames.

The type of interaction was then determined. Transient adhesion occurred when a *Bb* adhered to the microvessel and then subsequently detached and followed the direction of flow. Extravasation occurred when a *Bb* is seen crossing the microvessel into the surrounding extracellular matrix (ECM). An unknown outcome occurred when a *Bb* remained adhered to the microvessel at the last frame of the time-lapse. We assume that *Bb* with an unknown outcome will eventually detach or extravasate, and the outcome is unknown due to the imaging period ending. *Bb* initially present in the first frame of the time-lapse were excluded from analysis.

After each hour of perfusion, adhered *Bb* was manually counted. Extravasated *Bb* was quantified by taking a one-minute time-lapse at each location focused at the equatorial plane of the microvessel and counting the number of *Bb* that appears in the surrounding extracellular matrix (ECM). After perfusion with *Bb* or vehicle (co-culture media), functional assays were immediately performed (permeability, immune cell adhesion, and/or immunocytochemistry).

Glycocalyx Degradation

To degrade the glycocalyx, microvessels were perfused with a mix of 50 mU mL^{-1} of heparinase III (Millipore Sigma, H8891) and 20 mU mL^{-1} sialidase (Millipore Sigma, 10269611001) diluted in cell culture medium for 48 hours beginning immediately after seeding at 37 ˚C.

To quantify the expression level of the glycocalyx components, confocal z-stacks (1 µm thickness) were acquired at 40x magnification on a spinning disk confocal microscope system (Nikon Yokogawa X1) and illumination was provided by a LUNF laser fixture (Nikon). Maximal intensity projection fluorescence images of z-stacks of 50 slices were created for each polar side of the microvessel in Image J. Mean fluorescence intensity across the ROI was then recorded and normalized to the average fluorescence intensity of the DAPI nuclear stain of the projection.

TNF-α Treatment
TNF-α treatment was done by perfusing microvessels with 5 ng mL^{-1} of human TNF-α recombinant protein (R&D Systems, 210-TA) diluted in cell culture medium for 12 hours prior to initial permeability assays performed before perfusion with *Bb* or vehicle (co-culture media).

Permeability
To assess barrier function, permeability assays were conducted 1 hour prior and immediately following perfusion with *Bb* or vehicle (co-culture media). iEC and iBMEC microvessels were perfused with 2 µM fluorescein-conjugated 2 MDa dextran (Thermo Fisher Scientific, cat. No. D7137) and 2 µM Alexa Fluor 647-conjugated 10 kDa dextran (Thermo Fisher Scientific, cat. No. D22914) respectively in PBS. Phase contrast (Nikon Eclipse TiE) and fluorescence images (Excelitas Technologies, X-Cite 120LED Boost), were captured at 10x magnification in a live cell chamber at 37 ˚C. Images were captured every 2 minutes at the microvessel equatorial plane 10 minutes before and 30 minutes after perfusion of the dye solution.

To quantify permeability, images were stabilized and cropped (ImageJ, NIH) to a 3 mm x 0.6 mm rectangle and sectioned into 3 regions: microvessel lumen, upper ECM, and lower ECM (3 mm x 0.09 mm, with the ECM regions 0.15 mm away from the lumen). Each of these regions were further sectioned into 10 regions of interest (0.3 mm x 0.09 mm), and the integrated pixel density for each ROI over time was plotted. Permeability was then calculated using $P = (r/2)(1/\Delta I)(dI/dt)$ where r is the microvessel radius, ΔI is the increase in the lumen fluorescence intensity following injection of dye solution, and dI/dt is the rate of fluorescence intensity increase in the ECM.

Immune Cell Adhesion
Immune cell adhesion assays were conducted immediately following post *Bb* or vehicle (co-culture media) perfusion permeability assays. THP-1 (monocyte-like, ATCC, TIB-202) and HL-60 (neutrophil-like, ATCC CCL-240) cells were cultured in RPMI-1640 medium (Thermo Fisher Scientific, 11875093) supplemented with 10% fetal bovine serum (Sigma, F4135), and 1% penicillin-streptomycin (Thermo Fisher Scientific, 15140122). HL-60 culture medium was additionally supplemented with 1 mM sodium pyruvate (Thermo Fisher Scientific, 11360070). Both THP-1s and HL-60s were kept under passage 40 and maintained under a density of 2 x 10^6 cells mL^{-1}.

Prior to each experiment, THP-1s were stained with CellTracker deep red (Thermo Fisher Scientific, C34565) and HL-60s with Calcein-AM (Thermo Fisher Scientific, C1430) for 20 min according to product protocols. Cells were then washed twice with PBS before resuspending in co-culture media at a final concentration of 1 x 10^6 cells mL^{-1}. 70 µL of the cell suspension was then perfused through each microvessel for 10 min under low shear stress (~0.5 dyne cm^{-2}). Non-adherent cells were then washed by perfusing with 150 µL of co-culture media for 30 minutes. Firmly adherent THP-1s and HL-60s were manually counted for each microvessel and normalized by microvessel length. Immune cells were considered firmly adhered and included for quantification if it remained stationary across two sets of images taken two minutes apart. Fluorescence channels were used for quantification because the bright signal

allowed for cells out of the plane of focus to be detected and included, while immune cells out of focus under phase contrast would be difficult to count.

Immunocytochemistry and Image Analysis

Immunocytochemistry was performed following permeability assays done post *Bb* or vehicle (co-culture media) perfusion. Microvessels were fixed with 4% paraformaldehyde (Thermo Fisher Scientific, J19943.K2) overnight at 4 ˚C. Then, PBS was perfused at room temperature for three hours before blocking with 5% BSA (Jackson ImmunoResearch, 001-000-161) solution in PBS azide overnight at 4 ˚C.

Microvessels were then stained by: (1) perfusing with primary antibody solution anti-human ICAM-1 (Abcam, ab2213) or anti-human heparan sulfate (USBiological Life Sciences, H1890) at a 1:40 dilution overnight at 4 ˚C, (2) washing with PBS azide overnight at 4 ˚C, (3) perfusing with secondary antibody solution goat anti-mouse Alexa Fluor 647 (Thermo Fisher Scientific, A-21235) and/or Alexa Fluor 488 conjugated wheat germ agglutinin (Therm Fisher Scientific, W11261) at a 1:200 dilution for 40 min at room temperature, (4) washing with PBS azide overnight at 4 ^{o}C, and (5) perfusion with Fluoromount-G mounting medium with DAPI (Thermo Fisher Scientific, 00-4959-52) for 30 min at room temperature prior to imaging.

Confocal z-stacks (1 μm thickness) were acquired at 40x magnification on a spinning disk confocal microscope system (Nikon Yokogawa X1) and illumination was provided by a LUNF laser fixture (Nikon). To quantify the expression level of ICAM-1 for each individual cell, a maximal intensity projection of 50 slices were created for each polar side of the microvessel. Boundaries for each individual cell were then demarcated manually using the ZO-1 GFP fluorescence signal in Image J. The mean fluorescence intensity was then recorded. Only cells completely in the image were analyzed. Reported data was normalized to the average fluorescence intensity of the DAPI nuclear stain of the projection.

Borrelia burgdorferi proliferation and viability assay

Bb were inoculated at an initial density of 1 x 10^{6} cells mL^{-1} in BSK-H, EGM-2 or iBMEC maintenance media, or a mixture at ratios of 3 to 1, 1 to 1, or 1 to 3. *Bb* density and viability were assessed over 4 days. *Bb* density was quantified using a Petroff-Hausser bacteria counting chamber (Microscope World, HS-3900) with epifluorescence microscopy. *Bb* viability was assessed by morphology, where elongated are scored as viable, and rounded *Bb* scored as unviable.

Borrelia burgdorferi qPCR

Bb were first conditioned in 1 mL of each media condition at a density of 1 x 10^{7} cells mL^{-1} for 24 hours at 37 ˚C. To collect and isolate the RNA, *Bb* were then collected by centrifugation at 8000 x g for 5 minutes. The supernatant was then discarded, and the pellet was resuspended in 400 μL of Trizol (Thermo Fisher Scientific, 12183555). 80 μL of chloroform was then added and the mixture was vortexed and rested for 3 minutes. The mixture was then centrifuged at 12000 x g for 15 minutes, and the aqueous phase was transferred to a new Eppendorf tube. 200 uL of isopropanol was then added and incubated for 10 minutes at 4 ˚C. The mixture was then centrifuged again at 12000 x g for 10 minutes and the supernatant was discarded. The pellet was resuspended in 400 μL of 75% ethanol and vortexed. The pellet was then centrifuged at 7500 x g for 5 minutes, the supernatant was discarded, and the pellet was air dried for 10 minutes. The pellet was resuspended in 17.5 μL of RNase free water, 2 μL of DNase buffer, and 1 μL of DNaseI (New England BioLabs, M0303S) and incubated at 37 ˚C for 15 minutes.

cDNA reverse transcription was then performed on the isolated RNA. First, the RNA concentration was measured using a NanoDrop^{TM} 2000 Spectrophotometer (Thermo Fisher Scientific, ND2000CLAPTOP). 1.5 μg of RNA was used for each reaction. The cDNA master

mix was made by mixing 2 µL of 10X RT Buffer, 0.8 µL of 25X dNTP mix, 2 µL of 10X random primer, 1 µL of RT, and 4.2 µL of water for each reaction (Thermo Fisher Scientific, 4374966). 10 µL of cDNA master mix was added to 10 µL of the RNA and water mixture and loaded into a StepOnePlus™ Real-Time PCR System (Thermo Fisher Scientific, 4376600). The cycler then ran at 25 ˚C for 10 minutes, 37 ˚C for 120 minutes, and 85 ˚C for 5 minutes before holding at 4 ˚C.

qPCR was then performed on the cDNA. For each reaction, 100 ng of template RNA, 200 nM of each primer (forward and reverse), and 2X SYBR Green PCR Master Mix (Thermo Fisher Scientific, 4309155) was added into each well. The PCR system was then set to the following cycling parameter: 95 ˚C for 10 minutes, then 40 cycles of 95 ˚C for 15 seconds and 60 ˚C for 1 minute.

Borrelia burgdorferi 2D adhesion assay

To assess *Bb* adhesion after media conditioning, a 96 well glass bottom plate was first coated with 20 µg mL^{-1} fibronectin (Sigma-Aldrich, F2006) overnight at 37 ˚C. *Bb* were conditioned in each media condition for 24 hours at 37 ˚C. Each well was then washed with PBS and blocked with 1% BSA (Jackson ImmunoResearch, 001-000-161) for 2 hours. Each well was then washed again with PBS, and 5 x 10^5 *Bb* were added to each well for 4 hours at 37 ˚C. The *Bb* were then aspirated out, and each well was washed with PBS three times. Each well was then imaged using epifluorescence microscopy, and the number of viable adhered *Bb* was quantified and normalized to surface area imaged.

Borrelia burgdorferi motility assay

To assess the effect of co-culture media on *Bb* motility, 7 mg mL^{-1} neutralized collagen I solution (Corning, 354249) was first pipetted into each well of a 96-well cell culture plate and then gelled for 10 to 15 minutes at 37 ˚C. Then, media (BSK-H, 1:1 EGM-2 co-culture media, or 1:1 iBMEC co-culture media) was added to each well and placed in an incubator at 37 ˚C overnight. *Bb* were conditioned in each media condition for 24 hours at 37 ˚C. 1 x 10^6 *Bb* was then added to each well and incubated at 37 ˚C for three hours. Confocal time-lapses were acquired at 10x magnification on a spinning disk confocal microscope system (Nikon Yokogawa X1) and illumination was provided by a LUNF laser fixture (Nikon). Time-lapses were acquired over 5 minutes at an imaging frequency of 1 frame s^{-1}.

Statistics

Prism (GraphPad ver. 10) was used for conducting statistical analyses. All experimental values here are reported as mean with error bars denoting standard deviation (S.D.). Student's unpaired t-test (two-tailed with unequal variance) was used for comparison of two groups, while analysis of variance (ANOVA) was used for comparison of three or more groups. For three or more groups of experimental conditions, Tukey's multiple comparisons test was used, and p-values were multiplicity adjusted. Statistically significant differences were defined as $p < 0.05$ with the thresholds: * $p < 0.05$, ** $p < 0.01$, *** $p < 0.001$.

References

1. Revel, A.T., A.M. Talaat, and M.V. Norgard, *DNA microarray analysis of differential geneexpression inBorrelia burgdorferi, the Lymedisease spirochete.* PNAS, 2002.
2. Tokarz, R., et al., *Combined effects of blood and temperature shift on Borrelia burgdorferi gene expression as determined by whole genome DNA array.* Infect Immun, 2004. **72**(9): p. 5419-32.
3. Coburn, J., et al., *Lyme Disease Pathogenesis.* Curr Issues Mol Biol, 2021. **42**: p. 473-518.
4. Zhang, X., et al., *BB0323 function is essential for Borrelia burgdorferi virulence and persistence through tick-rodent transmission cycle.* J Infect Dis, 2009. **200**(8): p. 1318-30.
5. Li, X., et al., *Borrelia burgdorferi lacking BBK32, a fibronectin-binding protein, retains full pathogenicity.* Infect Immun, 2006. **74**(6): p. 3305-13.
6. Liang, F.T., et al., *Borrelia burgdorferi changes its surface antigenic expression in response to host immune responses.* Infect Immun, 2004. **72**(10): p. 5759-67.
7. Sumpio, B.E., J.T. Riley, and A. Dardik, *Cells in focus: endothelial cell.* The international journal of biochemistry & cell biology, 2002. **34**(12): p. 1508-1512.
8. Ebady, R., et al., *Biomechanics of Borrelia burgdorferi Vascular Interactions.* Cell Rep, 2016. **16**(10): p. 2593-2604.
9. Moriarty, T.J., et al., *Real-time high resolution 3D imaging of the lyme disease spirochete adhering to and escaping from the vasculature of a living host.* PLoS Pathog, 2008. **4**(6): p. e1000090.
10. Moriarty, T.J., et al., *Vascular binding of a pathogen under shear force through mechanistically distinct sequential interactions with host macromolecules.* Mol Microbiol, 2012. **86**(5): p. 1116-31.
11. Niddam, A.F., et al., *Plasma fibronectin stabilizes Borrelia burgdorferi-endothelial interactions under vascular shear stress by a catch-bond mechanism.* Proc Natl Acad Sci U S A, 2017. **114**(17): p. E3490-E3498.
12. Norman, M.U., et al., *Molecular mechanisms involved in vascular interactions of the Lyme disease pathogen in a living host.* PLoS Pathog, 2008. **4**(10): p. e1000169.
13. Bista, S., et al., *A novel laminin-binding protein mediates microbial-endothelial cell interactions and facilitates dissemination of Lyme disease pathogens.* The Journal of Infectious Diseases, 2020. **221**(9): p. 1438-1447.
14. Yang, X., et al., *Identification, characterization, and expression of three new members of the Borrelia burgdorferi Mlp (2.9) lipoprotein gene family.* Infection and immunity, 1999. **67**(11): p. 6008-6018.
15. Kariu, T., et al., *BB0323 and novel virulence determinant BB0238: Borrelia burgdorferi proteins that interact with and stabilize each other and are critical for infectivity.* The Journal of Infectious Diseases, 2015. **211**(3): p. 462-471.
16. Wang, K., et al., *Robust differentiation of human pluripotent stem cells into endothelial cells via temporal modulation of ETV2 with modified mRNA.* Science advances, 2020. **6**(30): p. eaba7606.
17. Lippmann, E.S., et al., *Derivation of blood-brain barrier endothelial cells from human pluripotent stem cells.* Nature biotechnology, 2012. **30**(8): p. 783-791.
18. Linville, R.M., et al., *Human iPSC-derived blood-brain barrier microvessels: validation of barrier function and endothelial cell behavior.* Biomaterials, 2019. **190**: p. 24-37.
19. Wong, A.D. and P.C. Searson, *Live-cell imaging of invasion and intravasation in an artificial microvessel platform.* Cancer research, 2014. **74**(17): p. 4937-4945.
